# Sterepinic Acids A–C, New Carboxylic Acids Produced by a Marine Alga-Derived Fungus

**DOI:** 10.3390/molecules23061336

**Published:** 2018-06-01

**Authors:** Takeshi Yamada, Miwa Matsuda, Mayuko Seki, Megumi Hirose, Takashi Kikuchi

**Affiliations:** Osaka University of Pharmaceutical Sciences, 4-20-1, Nasahara, Takatsuki, Osaka 569-1094, Japan; e12643@gap.oups.ac.jp (M.M.); e10118@gap.oups.ac.jp (M.S.); e11729@gap.oups.ac.jp (M.H.); t.kikuchi@gly.oups.ac.jp (T.K.)

**Keywords:** sterepinic acids, *Stereum* sp., marine microorganism, *Undaria pinnatifida*, vibralactones, phenylglycine methyl ester method

## Abstract

Sterepinic acids A–C (**1**–**3**), new carboxylic acids with two primary alcohols, have been isolated from a fungal strain of *Stereum* sp. OUPS-124D-1 attached to the marine alga *Undaria pinnatifida*. Dihydro-1,5-secovibralactone (**4**), a new vibralactone derivative, was isolated from the same fungal metabolites together with known vibralactone A (**5**), and 1,5-secovibralactone (**6**). The planar structures of these compounds have been elucidated by spectroscopic analyses using IR, HRFABMS, and NMR spectra. To determine the absolute configuration of the compounds, we used the phenylglycine methyl ester (PGME) method. These compounds exhibited less activity in the cytotoxicity assay against cancer cell lines.

## 1. Introduction

Our ongoing search for seeds of antitumor chemotherapy agents from marine microorganisms has led to the isolation of several antitumor and/or cytotoxic compounds [1,2,3,4,5,6,7,8]. In particular, we focused on the bioactive compounds with small molecular weight due to their advantages, such as easy synthesis and modification for increasing the activity. In addition, the synthesis of small bioactive compounds establishes a hypothetical biosynthesis mechanism of larger bioactive compounds. In this study, we isolated four new carboxylic acids with two primary alcohols, designated as sterepinic acids A–C (**1**–**3**) and dihydro-1,5-secovibralactone (**4**), together with the known vibralactone A (**5**) and 1,5-secovibralactone (**6**), from a strain of *Stereum* sp. OUPS-124D-1 derived from the marine alga *Undaria pinnatifida*. **5** was reported by Liu et al. [9], and many studies then followed this work, isolating the derivatives of **5** including **6** [10,11,12,13,14,15]. We report the determination of the absolute configurations of **1**–**4** by applying the phenylglycine methyl ester (PGME) method [16]. In addition, we report on the investigation of the cytotoxicity of these compounds against several cancer cell lines.

## 2. Results

**Figure 1:**
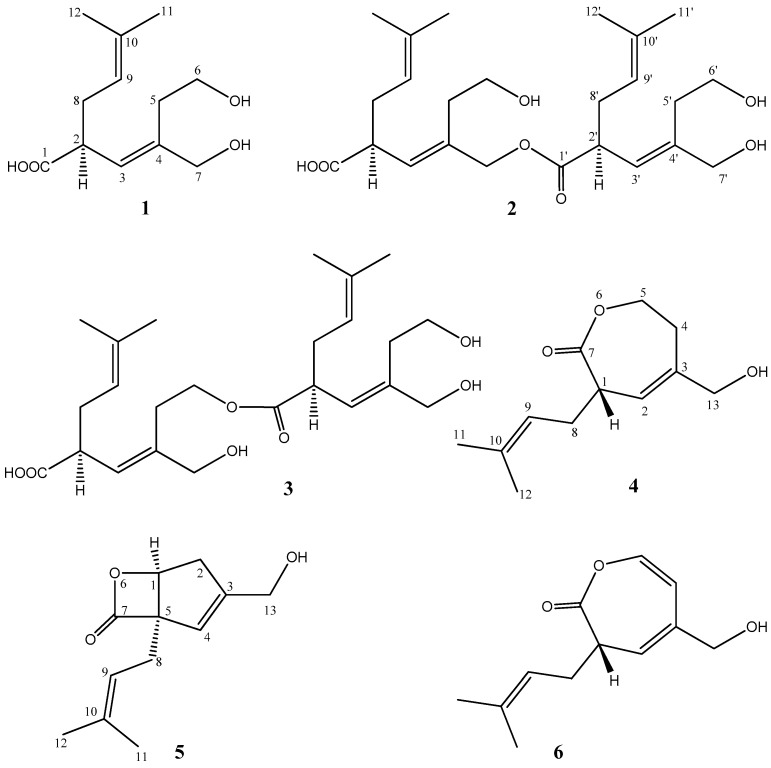
Structures of sterepinic acids A–C (**1**–**3**), dihydro-1,5-secovibralactone (**4**), and known compounds **5** and **6**.

*Stereum* sp., a microorganism from *U. pinnatifida*, was cultured at 27 °C for 5 weeks in a medium (50 L) containing 1% glucose, 1% malt extract, and 0.05% peptone in artificial seawater adjusted to pH 7.6. After the incubation, the culture was filtrated through DIAION HP-20, and its MeOH elution was purified employing a stepwise combination of silica gel column chromatography and reverse phase HPLC to afford sterepinic acids, A (**1**) (64.8 mg); B (**2**) (13.3 mg); C (**3**) (16.8 mg); and dihydro-1,5-secovibralactone (**4**) (12.4 mg), as a pale yellow oil, respectively (Figure 1). 

**Figure 2:**
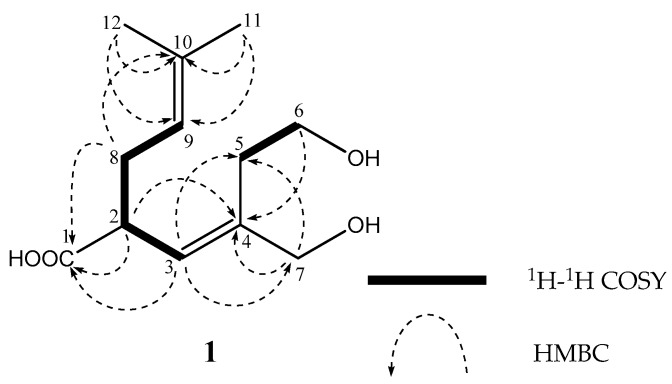
Selected ^1^H–^1^H COSY and HMBC correlations of **1**.

The molecular formula of sterepinic acid A (**1**) has been determined as C_12_H_20_O_4_ from its molecular weight of 229.1443 [M + H]^+^ in HRFABMS. Its IR spectrum exhibited bands at 3330 and 1710 cm^−1^, that are characteristics of hydroxy and carbonyl groups, respectively. An analysis of the ^1^H and ^13^C NMR spectra of **1** (Table 1 and Table S1), using DEPT and ^1^H–^13^C heteronuclear multiple quantum coherence spectroscopy (HMQC), showed the presence of two olefin methyls (C-11 and C-12); four sp^3^-hybridized methylenes (C-5, C-6, C-7, and C-8), including two oxygen-bearing sp^3^-methylenes (C-6 and C-7); one sp^3^-methine (C-2); two sp^2^-methines (C-3 and C-9); two quaternary sp^2^-carbons (C-4 and C-10); and one carbonyl group (C-1). In the ^1^H–^1^H correlation spectroscopy (COSY) analysis, correlations were observed between H-5 and H-6; H-2 and H-3; and H-2 and H-8, as shown by the bold lines in Figure 2. In the HMBC spectrum (Figure 2), the correlations from H-11 and H-12 to C-9 and C-10; from H-2 to C-1 and C-4; from H-3 to C-1, C-5, and C-7; from H-5 to C-3; from H-6 to C-4; from H-7 to C-3, C-4, and C-5; from H-8 to C-1 and C-10; from H-6 to C-4; and from H-7 to C-4, and C-5 elucidated the planar structure of **1** as 6-hydroxy-4-(hydroxymethyl)-2-(3-methylbut-2-en-1-yl) hex-3-enoic acid. The elucidation of the absolute stereostructure of **1** is described below, together with those of **2**–**4**.

**Figure 3:**
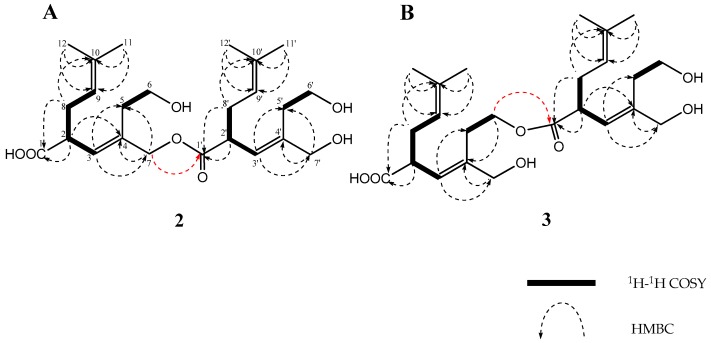
Selected ^1^H–^1^H COSY and HMBC correlations of **2** (**A**) and **3** (**B**).

Sterepinic acids, B (**2**) and C (**3**), were assigned the molecular formula of C_24_H_38_O_7_, with both compounds showing molecular weight almost twice as large as that of **1**. While the general features of NMR spectra (Table 1, Tables S2 and S3) closely resembled those of **1**, the ^1^H and ^13^C signals of **2** and **3** were observed in pairs or with the overlapping of two signals for each functional group (*vide info.*), except for the proton signal of the oxygen-bearing methylenes (C-7 (***δ***_H_ 4.48 d, and ***δ***_H_ 4.62 d) in **2**) and C-6 (***δ***_H_ 4.20 m) in **3**). This phenomenon suggested that **2** and **3** were the dimers of **1**. As expected, for the HMBC spectrum of **2** (Table S2), the correlations shown in Figure 3A were used to construct two carboxylic acids, both of which are identical to the planar structure of **1**. In addition, the correlation from H-7 in one carboxylic acid to C-1′ in another carboxylic acid revealed that the two carboxylic acids were condensed to a dimer esterified between C-7 and C-1′ (Figure 3A and Table S2). By contrast, the HMBC correlation from H-6 to C-1′observed in **3** demonstrated that the chemical structure of **3** was similar to that of the dimer esterified between C-6 and C-1′ (Figure 3B and Table S3). 

**Figure 4:**
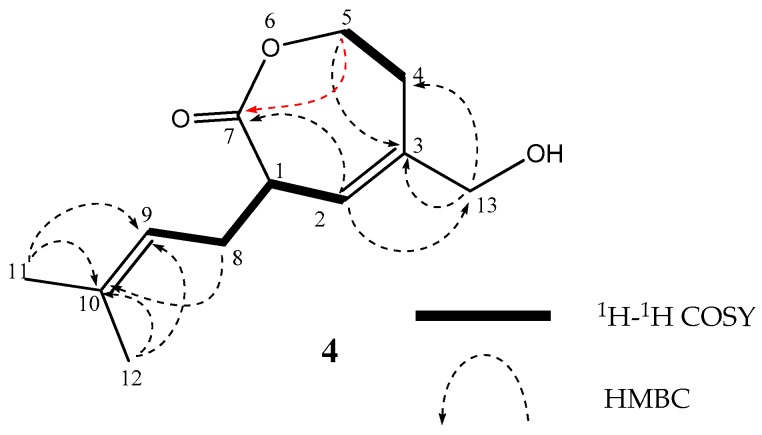
Selected ^1^H–^1^H COSY and HMBC correlations of **4**.

Dihydro-1,5-secovibralactone (**4**) exhibited the molecular formula C_12_H_20_O_4_, containing two fewer hydrogen atoms, and one less oxygen atom than **1**. Compared with the NMR spectra of **4** (Table 2 and Table S4), those of **1** showed large differences in the proton signals of H-1 (***δ***_H_ 3.68 m) and H-5 (***δ***_H_ 4.68 ddd and 4.33 ddd), corresponding to H-2 and H-6 in **1**, respectively, and the carbon signals of C-1 (***δ***_H_ 40.2), C-2 (***δ***_H_ 121.2), and C-7 (***δ***_H_ 174.3), corresponding to C-2, C-3, and C-1, respectively, in **1**. The numbering of the carbon positions followed the numbering mentioned in a previous report [6]. **4** was observed to be the monomer with the same carboxylic acid unit as **1**. In addition, HMBC correlations from H-5 to C-7 (Table S4 and Figure 4) elucidated the planar structure of **4** as a dihydro-isomer of 1,5-secovibralactone (**6**) [10].

**Figure 5:**
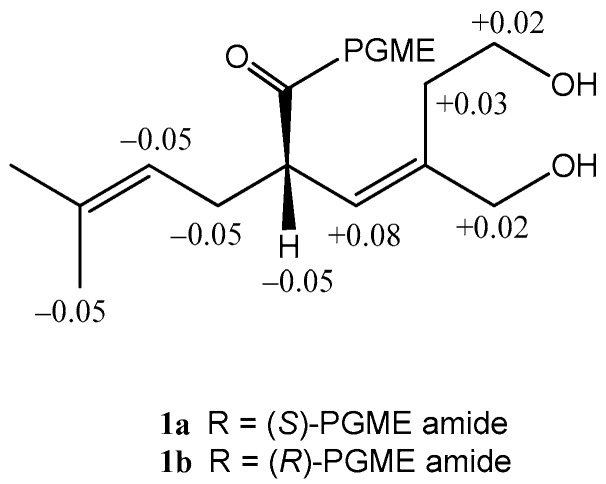
^1^H chemical-shift differences (Δ***δ*** ppm) between the (*S*)- and (*R*)- PGME amides **1a** and **1b**.

For the determination of the absolute stereostructures of metabolites isolated in this study, we first examined the absolute configuration of **1**, which is the common unit in all compounds of this study. **1** showed the presence of a secondary carboxy group at C-2, and we therefore used the PGME method [16]. The ^1^H chemical-shift differences between the (*S*)- and (*R*)-PGME amides **1a** and **1b** revealed the *S* configuration at C-2 (Figure 5).

**Scheme 1:**
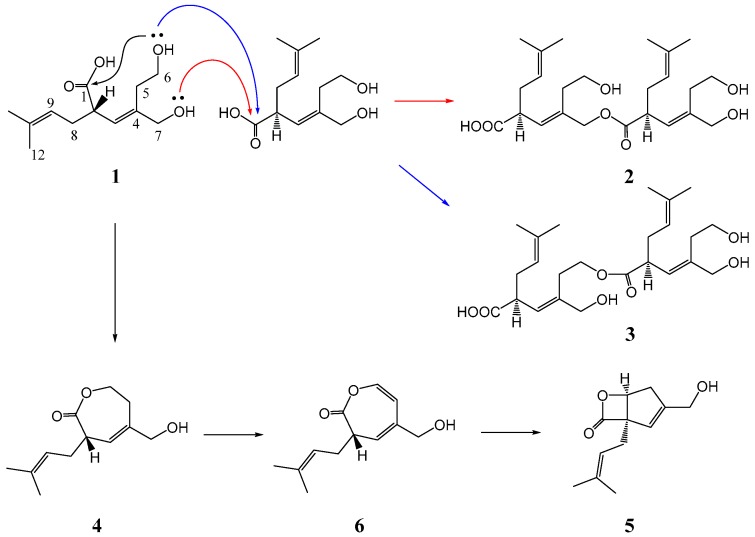
Plausible mechanism for the formation of **2**–**6** from **1**.

Next, for the elucidation of the stereochemistry of **2**–**4**, we attempted to perform hydrolysis to derive **1** from **2**–**4**; however, due to the small volume of reaction, the carboxylic acid was not produced. We therefore tried methanolysis to facilitate the purification of the product resulting from the reaction. The treatment with concd H_2_SO_4_ of MeOH solution of **2** only gave a methyl carboxylate, the spectral data (^1^H NMR spectrum and the optical rotation) for which were identical to those of the methyl ester of **1**; i.e., **2** is found to be in the 2*S*, 2′*S* absolute configuration. The same procedure applied to **3** and **4** revealed the *S* configuration at C-2 and C-2′ in **3**, and the *S* configuration at C-2 in **4**, respectively. This evidence confirmed that **2**–**4** were composed of **1**. A lone pair on the alcohol oxygen atom attacks a carboxy carbon atom by an intra- or intermolecular nucleophilic reaction, as shown by the arrows coded using three different colors (Scheme 1). The routes shown in red and blue, which are the dimerization routes, produce **2** and **3**, respectively. On the other hand, the route shown in black leads to **4** followed by a dehydrogenation to **6**. Meanwhile, Zhao et al., performed an in vitro enzymatic conversion, and verified biochemically the enzymatic production of **5** from **6** by the analyses of LC/MS/MS [17].

Cancer cell growth-inhibitory properties of sterepinic acids A–C (**1**–**3**) and dihydro-1,5-secovibralactone (**4**) were examined using murine P388 leukemia, human HL-60 leukemia, and murine L1210 leukemia cell lines; however, these metabolites did not exhibit significant activity against these cancer cells (Table 3). We therefore continue to investigate related compounds with more potent cytotoxicity from this fungal metabolite and examine another assay.

## 3. Materials and Methods 

### 3.1. General Experimental Procedures

NMR spectra were recorded on an Agilent-NMR-vnmrs (Agilent Technologies, Santa Clara, CA, USA) 600 MHz and 400 MHz with tetramethylsilane (TMS) as an internal reference. FABMS was recorded using a JEOL JMS-7000 mass spectrometer (JEOL, Tokyo, Japan). IR spectra was recorded on a JASCO FT/IR-680 Plus (Tokyo, Japan). Optical rotations were measured using a JASCO DIP-1000 digital polarimeter (Tokyo, Japan). DIAION HP20 (Mitsubishi Chemical, Tokyo, Japan), and Silica gel 60 (230–400 mesh, Nacalai Tesque, Inc. Kyoto, Japan) was used for column chromatography with medium pressure. ODS HPLC was run on a JASCO PU-1586 (Tokyo, Japan) equipped with a differential refractometer (RI-1531, Tokyo, Japan) and Cosmosil Packed Column 5C_18_-MSII (25 cm × 20 mm i.d., Nacalai Tesque, Inc., Kyoto, Japan). Analytical TLC was performed on precoated Merck aluminum sheets (DC-Alufolien Kieselgel 60 F254, 0.2 mm, Merck, Darmstadt, Germany) with the solvent system CH_2_Cl_2_–MeOH (19:1), and compounds were viewed under a UV lamp (AS ONE Co., Ltd., Osaka, Japan) and sprayed with 10% H_2_SO_4_ followed by heating.

### 3.2. Fungal Material

A strain of *Stereum* sp. was initially isolated from a piece of the marine alga *Undaria pinnatifida* collected at collected in Osaka bay, Japan in May 2015. The fungal strain was identified by Techno Suruga Laboratory Co., Ltd. The surface of the marine alga was wiped with EtOH, and its snip applied to the surface of nutrient agar layered in a Petri dish. Serial transfers of one of the resulting colonies provided a pure strain of *Stereum* sp.

### 3.3. Culturing and Isolation of Metabolites

The fungal strain was cultured at 27 °C for 4 weeks in a liquid medium (50 L) containing 1% malt extract, 0.05% peptone, and 1% d-glucose in artificial seawater adjusted to pH 7.5. The culture was filtered under suction, and the culture filtrate was passed through to DIAION HP20, and washed with water to remove water-soluble component. The fraction eluted with MeOH were evaporated in vacuo to afford a mixture of crude metabolites (10.2 g) that exhibited cytotoxicity against the P388 cell line (IC_50_ < 10 µg/mL). The mixture was chromatographed on a silica gel column with a CH_2_Cl_2_–MeOH gradient as the eluent to afford Fraction (Fr.) 1 (2% MeOH in CHCl_3_ eluate, 270.5 mg) and Fr. 2 (10% MeOH in CHCl_3_ eluate, 840.3 g). Fr. 1 was purified by ODS HPLC using MeOH–H_2_O (50:50) as the eluent to afford **4** (12.4 mg). Fr. 2 was purified by HPLC using MeOH–H_2_O (60:40) as the eluent to afford **2** (13.3 mg), **3** (16.8 mg), and Fr. 3 (102.3 mg). Fr. 3 was purified by ODS HPLC using MeOH–H_2_O (40:60) as the eluent to afford **1** (64.8 mg). 

Sterepinic acids A (**1**): Pale yellow oil; [α]_D_^22^ +58.0 (*c* 0.34, MeCN); IR (neat) ν_max_ / cm^−1^: 3330, 1710. FABMS *m*/*z* (%): 229 ([M + H]^+^, 71.4%), 211 (87.4%), 143 (34.2%), 69 (100%). HRFABMS *m*/*z* 229.1443 [M + Na]^+^ (calcd for C_12_H_21_O_4_: 229.1440). ^1^H and ^13^C NMR data are listed in Table 1 and Table S1 (SI).

Sterepinic acids B (**2**): Pale yellow oil; [α]_D_^22^ +141.7 (*c* 0.27, MeCN); IR (neat) ν_max_ / cm^−1^: 3362, 1730. FABMS *m*/*z* (%): 439 ([M + H]^+^, 40.9%), 211 (93.5%), 69 (100%). HRFABMS *m*/*z* 439.2694 [M + H]^+^ (calcd for C_24_H_39_O_7_: 439.2695). ^1^H and ^13^C NMR data are listed in Table 1 and Table S2 (SI).

Sterepinic acids C (**3**): Pale yellow oil; [α]_D_^22^ +53.5 (*c* 0.16, MeCN); IR (neat) ν_max_ / cm^−1^: 3383, 1710. FABMS *m*/*z* (%): 439 ([M + H]^+^, 15.9%), 211 (54.0%), 69 (96.1%). HRFABMS *m*/*z* 439.2694 [M + H]^+^ (calcd for C_24_H_39_O_7_: 439.2695). ^1^H and ^13^C NMR data are listed in Table 1 and Table S3 (SI).

Dihydro-1,5-secovibralactone (**4**): Pale yellow oil; [α]_D_^22^ +7.9 (*c* 0.32, MeCN); IR (neat) ν_max_ / cm^−1^: 3396, 1736. FABMS *m*/*z* (%): 211 ([M + H]^+^, 100%) 142 (37.7%), 69 (54.1%. HRFABMS *m*/*z* 211.1342 [M + H]^+^ (calcd for C_12_H_19_O_3_: 211.1334). ^1^H and ^13^C NMR data are listed in Table 1 and Table S3 (SI).

### 3.4. Chemical Transformation

#### 3.4.1. Formation of the (*S*)- and (*R*)-PGME Amides 

To a solution of **1** (5.8 mg, 0.025 mmol) and (*S*)-PGME (0.054 mmol) in dry DMF (1 mL) was added to EDC-HCl (0.050 mmol), HOBt (0.050 mmol), and DMAP (catalysis volume). The reaction mixture was stirred at room temperature 2 hours. Water (1.0 mL) was added to the reaction mixture, and then extracted using CH_2_Cl_2_. The organic layer was evaporated under reduced pressure, and the residue was purified by HPLC using MeOH–H_2_O (50:50) as the eluent to afford (*S*)-PGME amide **1a** (0.9 mg, 0.0024 mmol) as a pale yellow oil. 

**1** (6.7 mg, 0.030 mmol) and (*R*)-PGME (0.052 mmol) were treated with the same procedure to afford (*R*)-PGME amide **2a** (3.1 mg, 0.0083 mmol) as a pale yellow oil.

PGME amide **1a**: Pale yellow oil; HRFABMS *m*/*z* 376.2126 [M + H]^+^ (calcd for C_21_H_30_NO_5_: 376.2124). ^1^H NMR ***δ*** ppm (400 MHz in CDCl_3_): 1.61 (3H, s, H-11), 2.16 (1H, ddd, *J* 17.2, 7.6, 7.6 Hz, H-8A), 2.25 (1H, ddd, *J* 14.8, 6.0, 6.0 Hz, H-5A), 2.47 (1H, ddd, *J* 17.2, 7.6, 7.6 Hz, H-8B), 2.60 (1H, ddd, *J* 14.8, 7.6, 7.6 Hz, H-5B), 3.22 (1H, ddd, *J* 10.4, 7.6, 7.6 Hz, H-2), 3.68 (3H, s, OCH_3_), 3.79 (2H, m, H-6), 4.07 (1H, d, *J* 17.6 Hz, H-7A), 4.11 (1H, d, *J* 17.6 Hz, H-7B), 4.99 (1H, dd, *J* 7.6, 7.6 Hz, H-9), 5.54, (1H, d, *J* 8.0 Hz, Gly-CH), 5.60 (1H, d, *J* 10.4 Hz, H-3), 7.24-7.34 (5H, m, Ar.H).

PGME amide **1b**: Pale yellow oil; HRFABMS *m*/*z* 376.2126 [M + H]^+^ (calcd for C_21_H_30_NO_5_: 376.2124). ^1^H NMR ***δ*** ppm (400 MHz in CDCl_3_): 1.58 (3H, s, H-12), 1.64 (3H, s, H-11), 2.21 (1H, ddd, *J* 17.2, 7.6, 7.6 Hz, H-8A), 2.30 (1H, ddd, *J* 10.8, 5.6, 4.0 Hz, H-5A), 2.51 (1H, ddd, *J* 17.2, 7.6, 7.6 Hz, H-8B), 2.57 (1H, ddd, *J* 10.8, 8.0, 4.0 Hz, H-5B), 3.27 (1H, ddd, *J* 9.6, 7.6, 7.6 Hz, H-2), 3.69 (3H, s, OCH_3_), 3.77 (2H, m, H-6), 4.05 (1H, m, H-7A), 4.09 (1H, m, H-7B), 5.03 (1H, dd, *J* 7.6, 7.6 Hz, H-9), 5.54, (1H, d, *J* 8.0 Hz, Gly-CH), 5.52 (1H, d, *J* 9.6 Hz, H-3), 7.21–7.35 (5H, m, Ar.H).

#### 3.4.2. Formation of Methyl Ester of **1**

**1** (8.8 mg) was added trimethylsilyldiazomethane (10% in hexane) 2mL, and the reaction mixture was stirred at room temperature overnight. The reaction mixture was evaporated under reduced pressure, and the residue was purified by HPLC using MeOH–H_2_O (60:40) as the eluent to afford methyl ester (6.5 mg) as a pale yellow oil.

Methyl ester of **1**: Pale yellow oil; [α]_D_^22^ −7.9 (*c* 0.25, MeCN); HRFABMS *m*/*z* 243.1597 [M + H]^+^ (calcd for C_13_H_23_O_4_: 243.1597). ^1^H NMR ***δ*** ppm (600 MHz in CDCl_3_): 1.61 (3H, s, H-12), 1.68 (3H, s, H-11), 2.19 (1H, ddd, *J* 14.4, 7.2, 7.2 Hz, H-8A), 2.42 (1H, m, H-5A), 2.45 (1H, m, H-8B), 2.47 (1H, m, H-5B), 3.32 (1H, ddd, *J* 9.6, 7.8, 7.8 Hz, H-2), 3.64 (3H, s, OCH_3_), 3.73 (2H, m, H-6), 4.08 (1H, d, *J* 17.6 Hz, H-7A), 4.10 (1H, d, *J* 17.6 Hz, H-7B), 5.04 (1H, dd, *J* 7.2, 7.2 Hz, H-9), 5.56, (1H, d, *J* 10.2 Hz, H-3).

#### 3.4.3. Methanolysis of **2**–**4**

To a solution of **2** (3.2 mg) in MeOH (0,5 mg) was added concd H_2_SO_4_ (0.01 mL), and the reaction mixture was left at room temperature for 1 hr. The mixture was diluted with water, and extracted with CH_2_Cl_2_, and the extract was evaporated under reduced pressure, and then the residue was purified by HPLC using MeOH–H_2_O (60:40) as the eluent to afford methyl ester (0.8 mg) as a pale yellow oil.

Using the same procedure as above with **2**, a solution of **3** (3.3 mg) in MeOH (0.5 mL) was treated with concd H_2_SO_4_ (0.01 mL), and purified by HPLC using MeOH–H_2_O (60:40) as the eluent to afford methyl ester (0.8 mg).

Using the same procedure as above with **2**, a solution of **4** (2.4 mg) in MeOH (0.5 mL) was treated with concd H_2_SO_4_ (0.01 mL), and purified by HPLC using MeOH–H_2_O (60:40) as the eluent to afford methyl ester (0.7 mg).

## 4. Conclusions

In this study, new carboxylic acids designated as sterepinic acids A–C (**1**–**3**) and dihydro-1,5-secovibralactone (**4**), have been isolated from a strain of *Stereum* sp. derived from marine sponge. Their absolute configurations were established by the application of the PGME method to **1** and the chemical transformation of **2**–**4**. 

In the screening for the search of the seeds of antitumor agents, these compounds did not exhibit significant cytotoxic activity against three cancer cell lines. 

## Figures and Tables

**Table 1:** ^1^H and ^13^C NMR spectral data for metabolites (**1**–**3**) in CDCl_3._

Position	1				2				3				
	*δ*_H_ *^a^*		*δ*_C_		*δ*_H_ *^a^*		*δ*_C_		*δ*_H_ *^a^*			*δ*_C_	
1			177.5	(s)			173.5	(s)			174.3		(s)
2	3.27	m	44.9	(d)	3.28	m	45.4	(d)	3.28	m	44.9		(d)
3	5.50	d (10.2)	127.0	(d)	5.49	d (10.8)	129.3	(d)	5.55	d (9.6)	129.3		(d)
4			138.7	(s)			133.9	(s)			137.9		(s)
5A	2.25	m	31.9	(t)	2.18	m	32.3	(t)	2.30	ddd (14.4, 5.4, 5.4)	27.7		(t)
5B	2.49	m			2.54	m			2.54	ddd (14.4, 5.4, 5.4)			
6A	3.68	br s	61.0	(t)	3.65	br s	61.4	(t)	4.20	m	63.5		(t)
6B			66.8	(t)	3.72	br s							
7A	4.03	br s			4.05	d (13.2)	67.9	(t)	4.07	m	66.5		(t)
7B						d (13.2)							
8A	2.20	m	30.9	(t)	2.20	m	30.8 *^b^*^5^	(t)	2.20	m	31.4		(t)
8B	2.44	m			2.46	m			2.44	m			
9	5.04	dd	120.2	(d)	5.03	m	120.2 *^b^*^6^	(d)	5.02 *^b^*^1^	dd (7.2, 7.2)	120.2 *^b^*^2^		(d)
10			134.1	(s)			134.2 *^b^*^7^	(s)			134.3		(s)
11	1.67	s	25.7	(q)	1.67	s	25.7	(q)	1.67	s	25.7		(q)
12	1.60	s	17.8	(q)	1.60	s	17.8	(q)	1.59 *^b^*^3^	s	17.8 *^b^*^4^		(q)
1′							173.5	(s)			174.3		(s)
2′					3.28	m	45.4	(d)	3.28	m	44.9		(d)
3′					5.52	d (10.8)	127.2	(d)	5.51	d (9.6)	127.2		(d)
4′							139.6	(s)			138.7		(s)
5′A					2.29	m	32.3	(t)	2.25	m	32.2		(t)
5′B					2.54	m			2.51	m			
6′A					3.72	br s	60.5	(t)	3.65	br s	61.1		(t)
6′B									3.71	br s			
7′					4.05	br s	67.5	(t)	4.02	m	67.4		(t)
8′A					2.20	m	30.6 *^b^*^5^	(t)	2.20	m	31.4		(t)
8′B					2.46	m			2.44	m			
9′					5.03	m	120.3 *^b^*^6^	(d)	5.06 *^b^*^1^	dd (7.2, 7.2)	120.3 *^b^*^2^		(d)
10′							134.3 *^b^*^7^	(s)			134.3		(s)
11′					1.67	s	25.7	(q)	1.67	s	25.7		(q)
12′					1.60	s	17.8	(q)	1.61 *^b^*^3^	s	17.9 *^b^*^4^		(q)

*^a^*^1^H chemical shift values (*δ* ppm from SiMe_4_) followed by multiplicity. *^b^*^1–*b*7^ interchangeable.

**Table 2:** ^1^H and ^13^C NMR spectral data for metabolites **4** in CDCl_3._

Position	4			
	*δ*_H_ *^a^*		*δ*_C_	
1	3.68	m	40.2	(d)
2	5.36	br s	121.2	(d)
3			139.2	(s)
4A	2.45	br d (19.2)	30.3	(t)
4B	2.59	m		
5α	4.68	ddd (12.6, 12.6, 1.8)	64.4	(t)
5β	4.33	ddd (12.6, 4.8, 2.4)		
6				
7			174.3	(s)
8A	2.33	ddd (14.4, 6.6, 6.6)	30.1	(t)
8B	2.52	ddd (14.4, 6.6, 6.6)		
9	5.14	dd (6.6, 6.6)	120.9	(d)
10			134.6	(s)
11	1.72	s	25.8	(q)
12	1.67	s	18.0	(q)
13A	3.99	d (13.8)	67.4	(t)
13B	4.01	d (13.8)		

*^a^* As in Table 1.

**Table 3:** Cytotoxicity assay against P388, HL-60, and L1210.

Compounds	Cell Line P388	Cell Line HL-60	Cell Line L1210
	IC_50_ (μM) *^a^*	IC_50_ (μM) *^a^*	IC_50_ (μM) *^a^*
**1**	>500	>500	>500
**2**	>500	236.7	>500
**3**	>500	60.2	480.9
**4**	>500	189.5	>500
5-fluorouracil *^b^*	6	4.9	4.5

*^a^* DMSO was used as vehicle. *^b^* Positive control.

## Supplementary Materials

The following are available online, Table S1: Spectral data including 2D NMR data for **1**, Table S2: Spectral data including 2D NMR data for **2**, Table S3: Spectral data including 2D NMR data for **3**, Table S4: Spectral data including 2D NMR data for 4, Figure S1: ^1^H NMR spectra of **1** in CDCl_3_, Figure S2: ^13^C NMR spectra of **1** in CDCl_3_, Figure S3: ^1^H-^1^H COSY of **1**, Figure S4: NOESY of **1**, Figure S5: HMQC of **1**, Figure S6: HMBC of **1**, Figure S7: ^1^H NMR spectrum of **2** in CDCl_3_, Figure S8: ^13^C NMR spectrum of **2** in CDCl_3_, Figure S9: ^1^H-^1^H COSY of **2**, Figure S10: NOESY of **2**, Figure S11: HMQC of **2**, Figure S12: HMBC of **2**, Figure S13: ^1^H NMR spectrum of **3** in CDCl_3_, Figure S14: ^13^C NMR spectrum of **3** in CDCl_3_, Figure S15: ^1^H-^1^H COSY of **3**, Figure S16: NOESY of **3**, Figure S17: HMQC of **3**, Figure S18: HMBC of **3**, Figure S19: ^1^H NMR spectrum of **4** in CDCl_3_, Figure S20: ^13^C NMR spectrum of **4** in CDCl_3_, Figure S21: ^1^H-^1^H COSY of **4**, Figure S22: NOESY of **4**, Figure S25: HMQC of **4**, Figure S24: HMBC of **4**, Figure S25: ^1^H NMR spectra of **1a** in CDCl_3_, Figure S26: ^1^H NMR spectra of **1b** in CDCl_3_, Figure S27: ^1^H NMR spectra of methyl ester of **1** in CDCl_3_.

## References

[1] Muroga Y., Yamada T., Numata A., Tanaka R. (2009). Chaetomugilins I–O, new potent cytotoxic metabolites from a marine-fish-derived Chaetomium species. Stereochemistry and biological activities. Tetrahedron.

[2] Yamada T., Kitada H., Kajimoto T., Numata A., Tanaka R. (2010). The relationship between the CD Cotton effect and the absolute configuration of FD-838 and its seven stereoisomers. J. Org. Chem..

[3] Yamada T., Kikuchi T., Tanaka R., Numata A. (2012). Halichoblelides B and C, potent cytotoxic macrolides from a *Streptomyces* species separated from a marine fish. Tetrahedron Lett..

[4] Kitano M., Yamada T., Amagata T., Minoura K., Tanaka R., Numata A. (2012). Novel pyridinopyrone sesquiterpene type pileotin produced by a sea urchin-derived *Aspergillus* sp.. Tetrahedron Lett..

[5] Yamada T., Mizutani Y., Umebayashi Y., Inno N., Kawashima M., Kikuchi T., Tanaka R. (2014). A novel ketoaldehyde decalin derivative, produced by a marine sponge-derived *Trichoderma harzianum*. Tetrahedron Lett..

[6] Yamada T., Umebayashi Y., Kawashima M., Sugiura Y., Kikuchi T., Tanaka R. (2015). Determination of the chemical structures of tandyukisins B–D, isolated from a marine sponge-derived fungus. Mar. Drugs.

[7] Suzue M., Kikuchi T., Tanaka R., Yamada T. (2016). Tandyukisins E and F, novel cytotoxic decalin derivatives isolated from a marine sponge-derived fungus. Tetrahedron Lett..

[8] Yamada T., Suzue M., Arai T., Kikuchi T., Tanaka R. (2017). Trichodermanins C–E, new diterpenes with a fused 6-5-6-6 ring system produced by a marine sponge-derived fungus. Marine Drugs.

[9] Liu D.Z., Wang F., Liao T.G., Tang J.G., Steglich W., Zhu H.J., Liu J.K. (2006). Vibralactone: A lipase inhibitor with an unusual fused β-lactone produced by cultures of the basidiomycete. Boreostereum vibrans. Org. Lett..

[10] Jiang M.Y., Wang F., Yang X.L., Fang L.Z., Dong Z.J., Zhu H.J., Liu J.K. (2008). Derivatives of vibralactone from cultures of the basidiomycete *Boreostereum vibrans*. Chem. Pharm. Bull..

[11] Jiang M.Y., Zhang L., Dong Z.J., Yang Z.L., Leng Y., Liu J.K. (2010). Vibralactones D–F from cultures of the basidiomycete *Boreostereum vibrans*. Chem. Pharm. Bull..

[12] Ding J.H., Feng T., Li Z.H., Li L., Liu J.K. (2012). Twelve new compounds from the basidiomycete *Boreostereum vibrans*. Nat. Prod. Bioprospect..

[13] Wang G.Q., Wei K., Feng T., Li Z.H., Zhang L., Wang Q.A., Liu J.K. (2012). Vibralactones G-J from cultures of the basidiomycete *Boreostereum vibrans*. J. Asian Nat. Prod. Res..

[14] Wang G.Q., Wei K., Li Z.H., Feng T., Ding J.H., Wang Q.A., Liu J.K. (2013). Three new compounds from the cultures of basidiomycete *Boreostereum vibrans*. J. Asian Nat. Prod. Res..

[15] Chen H.P., Zhao Z.Z., Yin R.H., Yin X., Feng T., Li Z.H., Wei K., Liu J.K. (2014). Six new vibralactone derivatives from cultures of the fungus *Boreostereum vibrans*. Nat. Prod. Bioprospect..

[16] Yabuuchi T., Kusumi T. (2000). Phenylglycine Methyl Ester, a Useful Tool for Absolute Configuration Determination of various chiral carboxylic acids. J. Org. Chem..

[17] Zhao P.J., Yang Y.L., Du L., Liu J.K., Zeng Y. (2013). Elucidating the biosynthetic pathway for vibralactone: A pancreatic lipase inhibitor with a fused bicyclic β-lactone. Angew. Chem. Int. Ed..

