# Supplementary material for: Sterepinic Acids A–C, New Carboxylic Acids Produced by a Marine Alga-Derived Fungus
Source: Molecules. 2018 Jun 1;23(6):1336. doi: 10.3390/molecules23061336 (PMC6099533; doi:10.3390/molecules23061336)
Supplement: Supplementary file 1 [file molecules-23-01336-s001.zip › molecules-310462-SI.pdf]

Supplementary Information

Sterepinic acids A–C, new carboxylic acids produced by a marine alga-derived Fungus

Takeshi Yamada * Miwa Matsuda, Mayuko Seki, Megumi Hirose and Takashi Kikuchi

**Structures of metabolites in the fungal strain**	**2**
**Table S1 Spectral data including 2D NMR data for 1**	**3**
**Table S2 Spectral data including 2D NMR data for 2**	**4**
**Table S3 Spectral data including 2D NMR data for 3**	**5**
**Table S4 Spectral data including 2D NMR data for 4**	**6**
**Figure S1 ^{1}H NMR spectrum of 1 in $CDCl_3$**	**7**
**Figure S2 ^{13}C NMR spectrum of 1 in $CDCl_3$**	**8**
**Figure S3 ^{1}H-^{1}H COSY of 1**	**9**
**Figure S4 NOESY of 1**	**10**
**Figure S5 HMQC of 1**	**11**
**Figure S6 HMBC of 1**	**12**
**Figure S7 ^{1}H NMR spectrum of 2 in $CDCl_3$**	**13**
**Figure S8 ^{13}C NMR spectrum of 2 in $CDCl_3$**	**14**
**Figure S9 ^{1}H-^{1}H COSY of 2**	**15**
**Figure S10 NOESY of 2**	**16**
**Figure S11 HMQC of 2**	**17**
**Figure S12 HMBC of 2**	**18**
**Figure S13 ^{1}H NMR spectrum of 3 in $CDCl_3$**	**19**
**Figure S14 ^{13}C NMR spectrum of 3 in $CDCl_3$**	**20**
**Figure S15 ^{1}H-^{1}H COSY of 3**	**21**
**Figure S16 NOESY of 3**	**22**
**Figure S17 HMQC of 3**	**23**
**Figure S18 HMBC of 3**	**24**
**Figure S19 ^{1}H NMR spectrum of 4 in $CDCl_3$**	**25**
**Figure S20 ^{13}C NMR spectrum of 4 in $CDCl_3$**	**26**
**Figure S21 ^{1}H-^{1}H COSY of 4**	**27**
**Figure S22 NOESY of 4**	**28**
**Figure S23 HMQC of 4**	**29**
**Figure S24 HMBC of 4**	**30**
**Figure S25 ^{1}H NMR spectra of 1a in $CDCl_3$**	**31**
**Figure S26 ^{1}H NMR spectra of 1b in $CDCl_3$**	**35**
**Figure S27 ^{1}H NMR spectra of methyl ester of 1 in $CDCl_3$**	**38**

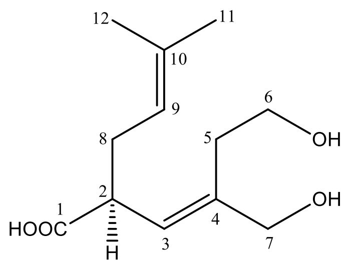

1

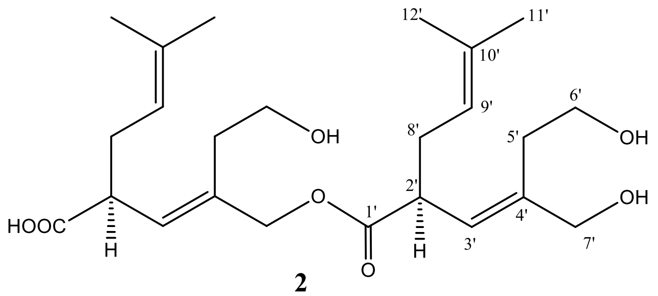

2

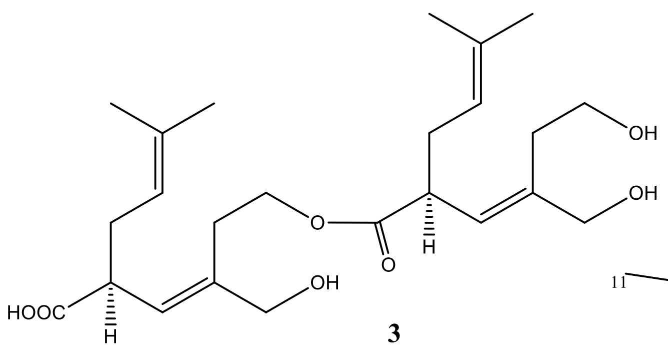

3

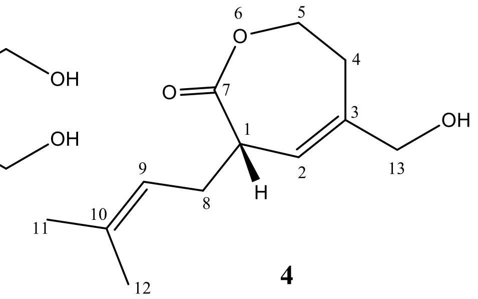

4

Table S1 NMR spectral data of **1** in $CDCl_3$

Position	δ_H[a]		J/Hz	^1H-^1H COSY	NOESY[b]	δ_C		HMBC (C)[c]
1						177.5	(s)	
2	3.27	m		3, 8A, 8B	9, 11, 12	44.9	(d)	1, 3, 4, 8, 9
3	5.50	d	10.2 (2)	2	8A, 8B, 9, 11, 12	127.0	(d)	1, 2, 5, 7, 8
4						138.7	(s)	
5A	2.25	m		5B, 6		31.9	(t)	3, 4, 6, 7
5B	2.49	m		5A, 6				3, 4, 6, 7
6	3.68	br s		5A, 5B	11, 12	61.0	(t)	4
7	4.03	br s			3, 11, 12	66.8	(t)	3, 4, 5
8A	2.20	m		2, 8B, 9	3, 11, 12	30.9	(t)	1, 2, 3, 9, 10
8B	2.44	m		2, 8A, 9	3, 11, 12			1, 2, 3, 9, 10
9	5.04	dd	7.2 (8), 7.2 (8)	8A, 8B	2, 3, 11, 12	120.2	(d)	2, 8, 11, 12
10						134.1	(s)	
11	1.67	s			2, 3, 6, 7, 8A, 8B, 9	25.7	(q)	9, 10, 12
12	1.60	s			2, 3, 6, 7, 8A, 8B, 9	17.8	(q)	9, 10, 11

a ^1H chemical shift values (δ ppm from $SiMe_4$) followed by multiplicity and then the coupling constants (*J* /Hz). Figures in parentheses indicate the proton coupling with that position. b The correlations with geminal and vicinal protones are removed. c Long range ^1H-^{13}C correlations from H to C observed in the HMBC experiment.

Table S2 NMR spectral data of **2** in $CDCl_3$

Position	δ_H[a]		J/Hz	^1H-^1H COSY	NOESY[b]	δ_C		HMBC (C)[c]
1						173.5	(s)	
2	3.28	m		3, 8A, 8B	5, 9	45.4	(d)	1, 3, 4, 8, 9
3	5.49	d	10.8 (2)	2	7A, 7B, 8A, 8B	129.3	(d)	2, 5, 7, 8
4						133.9	(s)	
5A	2.18	m		5B, 6A, 6B	2	32.3	(t)	3, 4, 6, 7
5B	2.54	m		5A, 6A, 6B	2			3, 4, 6, 7
6A	3.65	br s		5A, 5B, 6B		61.4	(t)	
6B	3.72	br s		5A, 5B, 6A				
7A	4.48	d	13.2 (7B)	7B	3	67.9	(t)	1, 3, 4, 5
7B	4.62	d	13.2 (7A)	7A	3			1, 3, 4, 5
8A	2.20	m		2, 8B, 9	3, 11, 12	30.8[d1]	(t)	1, 2, 3, 9, 10
8B	2.46	m		2, 8A, 9	3, 11, 12			1, 2, 3, 9, 10
9	5.03	m		8A, 8B	2, 11, 12	120.2[d2]	(d)	2, 8, 11, 12
10						134.2[d3]	(s)	
11	1.67	s			9	25.7	(q)	9, 10, 12
12	1.60	s			8A, 8B, 9	17.8	(q)	9, 10, 11
1'						173.5	(s)	
2'	3.28	m		3', 8'A, 8'B	5', 9'	45.4	(d)	1', 8', 9'
3'	5.52	d	10.2 (2')	2'	7', 8'A, 8'B	127.2	(d)	5', 7'
4'						139.6	(s)	
5'A	2.29	m		5'B, 6'	2'	32.3	(t)	3', 4', 6', 7'
5'B	2.54	m		5'A, 6'	2'			3', 4', 6', 7'
6'	3.72	br s		5'A, 5'B		60.5	(t)	
7'	4.05	br s			3'	67.5	(t)	3', 4', 5'
8'A	2.20	m		2', 8'B, 9'	3', 11', 12'	30.6[d1]	(t)	1', 2', 3', 9', 10'
8'B	2.46	m		2', 8'A, 9'	3', 11', 12'			1', 2', 3', 9', 10'
9'	5.03	m		8'A, 8'B	2', 11', 12'	120.3[d2]	(d)	2', 8', 11', 12'
10'						134.3[d3]	(s)	
11'	1.67	s			9'	25.7	(q)	9', 10', 12'
12'	1.60	s			8'A, 8'B, 9'	17.8	(q)	9', 10', 11'

a 1H che+B14:L36mical shift values (d ppm from SiMe4) followed by multiplicity and then the coupling constants (J/Hz). Figures in parentheses indicate the proton coupling with that position. b The correlations with geminal and vicinal protones are removed. c Long range 1H-13C correlations from H to C observed in the HMBC experiment. d1-d3 interchangeable

Table S3 NMR spectral data of **3** in $CDCl_3$

Position	δ_H[a]		J/Hz	^{1}H-^{1}H COSY	NOESY[b]	δ_C		HMBC (C)[c]
1						174.3	(s)	
2	3.28	m		3, 8A, 8B	5, 9	44.9	(d)	1, 8, 9
3	5.55	d	9.6 (2)	2	7, 9	129.3	(d)	5, 7
4						137.9	(s)	
5A	2.30	ddd	14.4 (5B), 5.4 (6), 5.4 (6)	5B, 6	2	27.7	(t)	3, 4, 6, 7
5B	2.54	ddd	14.4 (5B), 5.4 (6), 5.4 (6)	5A, 6	2			3, 4, 6, 7
6	4.20	m		5A, 5B		63.5	(t)	1', 4
7	4.07	m			3	66.5	(t)	3, 4
8A	2.20	m		2, 8B, 9	12	31.4	(t)	1, 2, 3, 9, 10
8B	2.44	m		2, 8A, 9	12			1, 2, 3, 9, 10
9	5.02[d1]	dd	7.2 (8A), 7.2 (8B)	8A, 8B	2, 3, 11	120.2[d2]	(d)	2, 8, 11, 12
10						134.3	(s)	
11	1.67	s			9	25.7	(q)	9, 10, 12
12	1.59[d3]	s			8A, 8B	17.8[d4]	(q)	9, 10, 11
1'						174.3	(s)	
2'	3.28	m		3', 8'A, 8'B	5', 9'	44.9	(d)	1', 3', 4', 8', 9'
3'	5.51	d	9.6 (2')	2'	7', 8'A, 8'B, 9'	127.2	(d)	1', 2', 5', 7', 8'
4'						138.7	(s)	
5'A	2.25	m		5'B, 6'A, 6'B	2'	32.2	(t)	3', 4', 6', 7'
5'B	2.51	m		5'A, 6'A, 6'B	2'			3', 4', 6', 7'
6'A	3.65	br s		5'A, 5'B, 6'B		61.1	(t)	
6'B	3.71	br s		5'A, 5'B, 6'A				
7'	4.02	m			3'	67.4	(t)	3', 4'
8'A	2.20	m		2', 8'B, 9'	12'	31.4	(t)	1', 2', 3', 9', 10'
8'B	2.44	m		2', 8'A, 9'	12'			1', 2', 3', 9', 10'
9'	5.06[d1]	dd	7.2 (8'A), 7.2 (8'B)	8'A, 8'B	2', 3', 11'	120.3[d2]	(d)	11', 12'
10'						134.3	(s)	
11'	1.67	s			9'	25.7	(q)	9', 10', 12'
12'	1.61[d3]	s			8'A, 8'B	17.9[d4]	(q)	9', 10', 11'

a 1H che+B23:L35mical shift values (d ppm from SiMe4) followed by multiplicity and then the coupling constants (J/Hz). Figures in parentheses indicate the proton coupling with that position. b The correlations with geminal and vicinal protones are removed. c Long range 1H-13C correlations from H to C observed in the HMBC experiment. d1-d4 interchangeable

Table S4 NMR spectral data of **4** in $CDCl_3$

Position	δ_H [a]		J/Hz	^1H-^1H COSY	NOESY [b]	δ_C		HMBC (C) [c]
1	3.68	m		2, 8A, 8B	9	40.2	(d)	
2	5.36	br s		1	8A, 8B, 9,13A, 13B	121.2	(d)	4, 7, 8, 13
3						139.2	(s)	
4A	2.45	br d	19.2 (5B)	4B, 5	13A	30.3	(t)	
4B	2.59	m		5A, 5B, 6B	13B			
5α	4.68	ddd	12.6 (4B), 12.6 (5β), 1.8 (4A)	4, 5β		64.4	(t)	3, 4, 7
5β	4.33	ddd	12.6 (5α), 4.8 (4B), 2.4 (4A)	4, 5α				3, 4, 7
6								
7						174.3	(s)	
8A	2.33	ddd	14.4 (8B), 6.6 (1), 6.6 (9)	1, 8B, 9	2, 11	30.1	(t)	1, 2, 7, 9, 10
8B	2.52	ddd	14.4 (8A), 6.6 (1), 6.6 (9)	1, 8A, 9	2, 11			1, 2, 7, 9, 10
9	5.14	dd	6.6 (8A), 6.6 (8B)	8A, 8B	1, 2, 11	120.9	(d)	1, 8, 11, 12
10						134.6	(s)	
11	1.72	s			9	25.8	(q)	9, 10, 12
12	1.67	s			8A, 8B	18.0	(q)	9, 10, 12
13A	3.99	d	13.8 (13B)		2, 4	67.4	(t)	2, 3, 4
13B	4.01	d	13.8 (13A)		2, 4			

a ^1H chemical shift values (δ ppm from $SiMe_4$) followed by multiplicity and then the coupling constants (*J*/Hz). Figures in parentheses indicate the proton coupling with that position. b The correlations with geminal and vicinal protones are removed. c Long range ^1H-^{13}C correlations from H to C observed in the HMBC experiment.

Figure S1 ^{1}H NMR spectrum of **1**

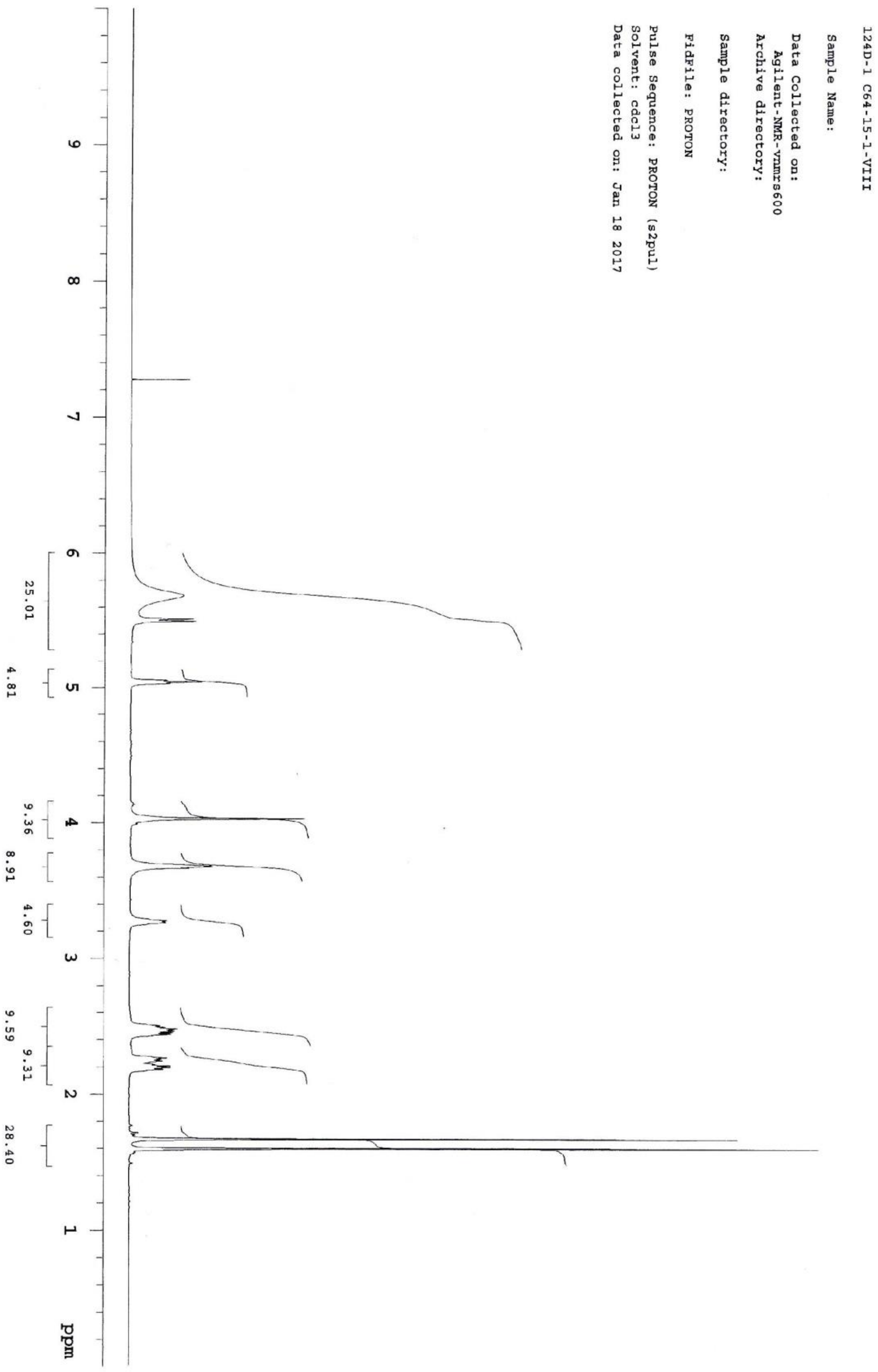

Figure S2 ^{13}C NMR spectrum of 1 in $CDCl_3$

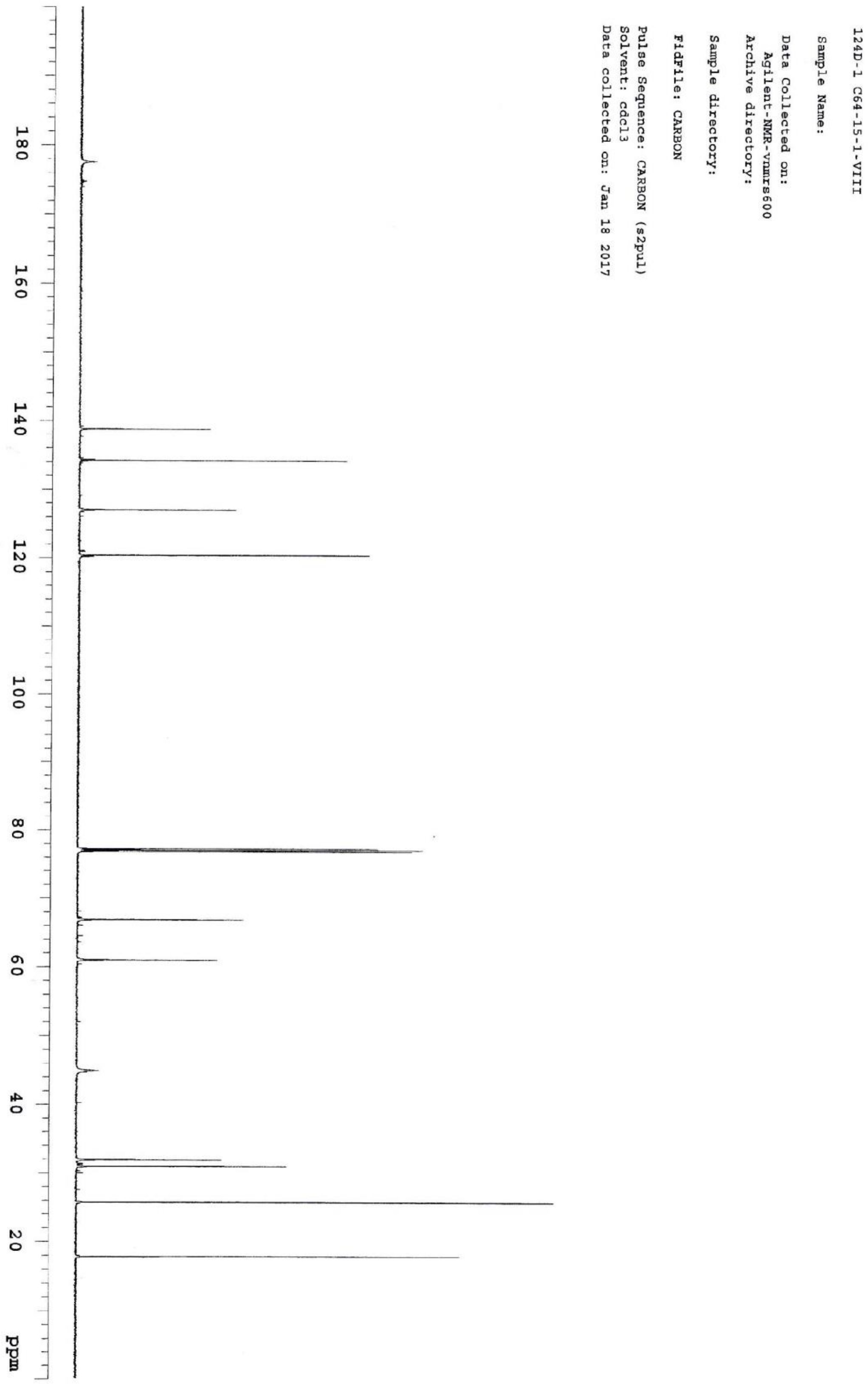

Figure S3 ^{1}H-^{1}H COSY of 1

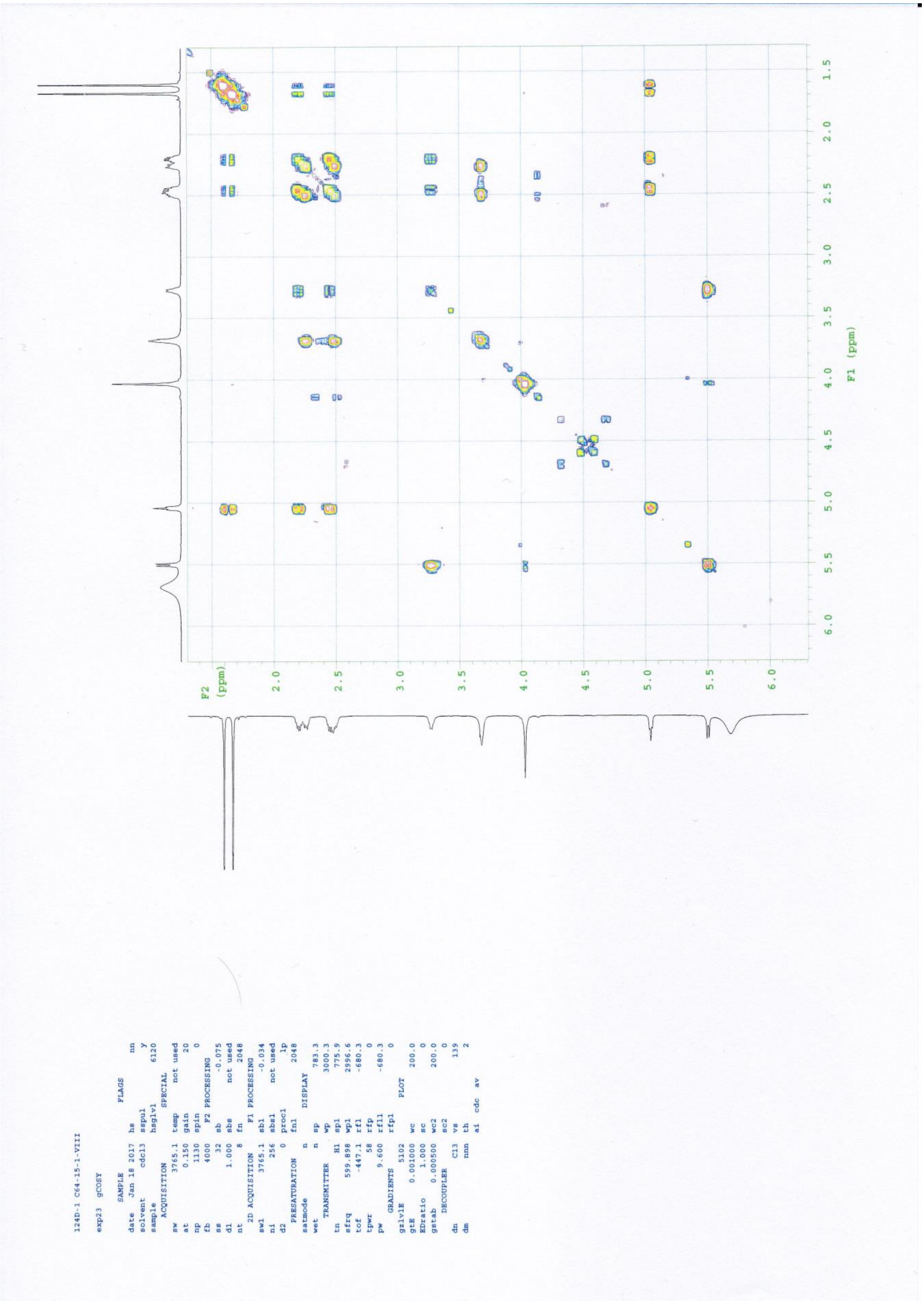

Figure S4　NOESY of 1

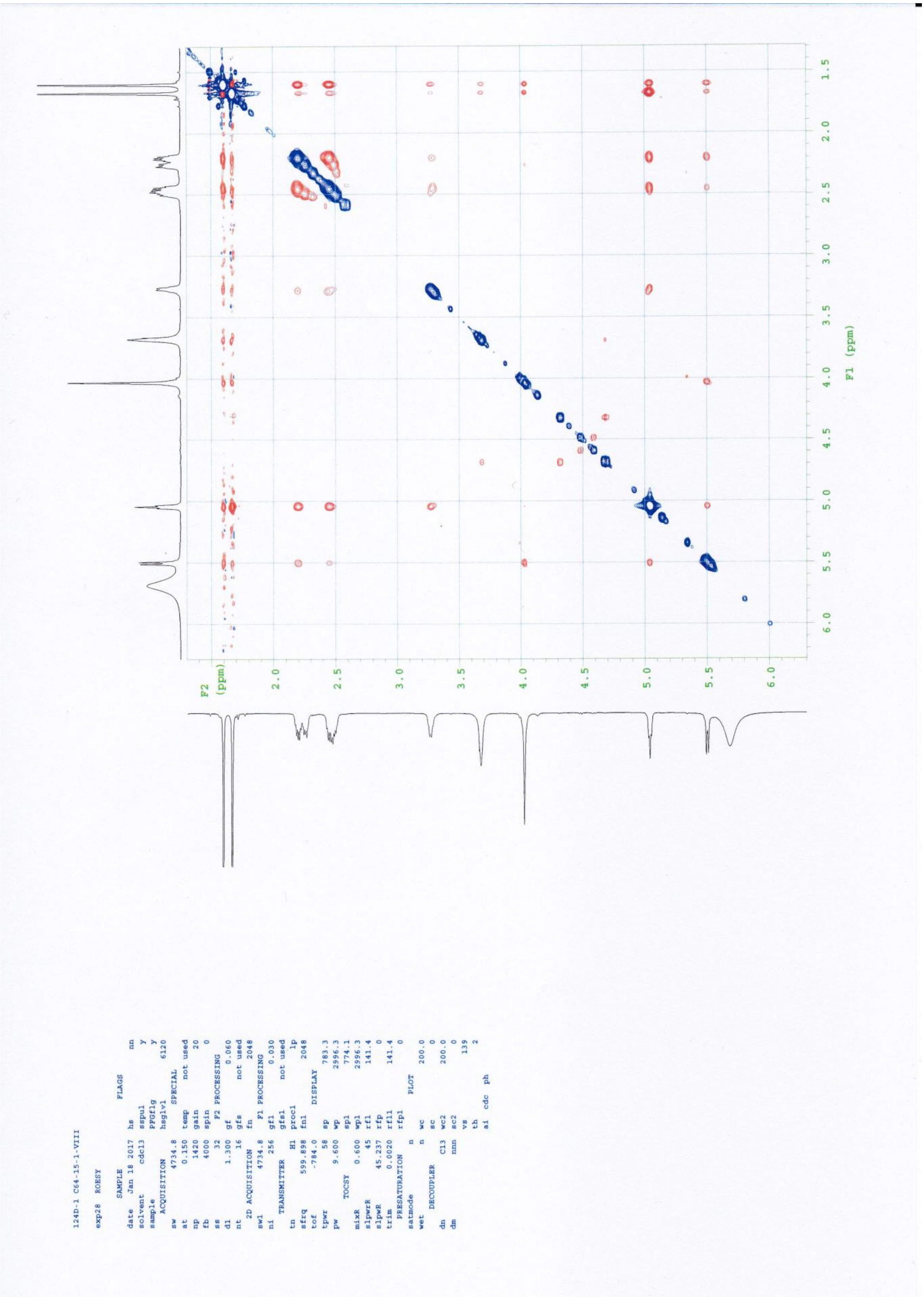

Figure S5 HMQC of 1

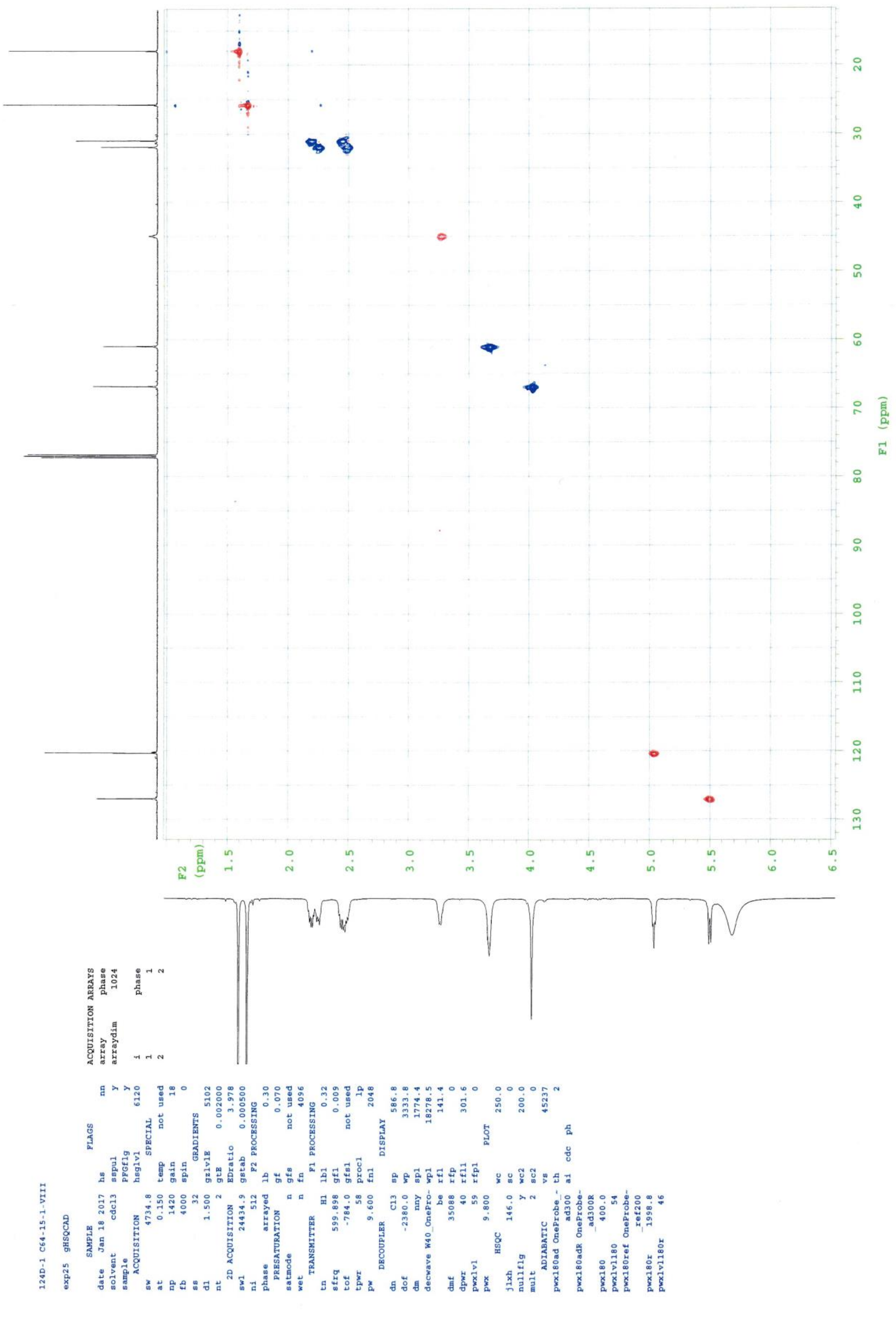

Figure S6 HMBC of 1

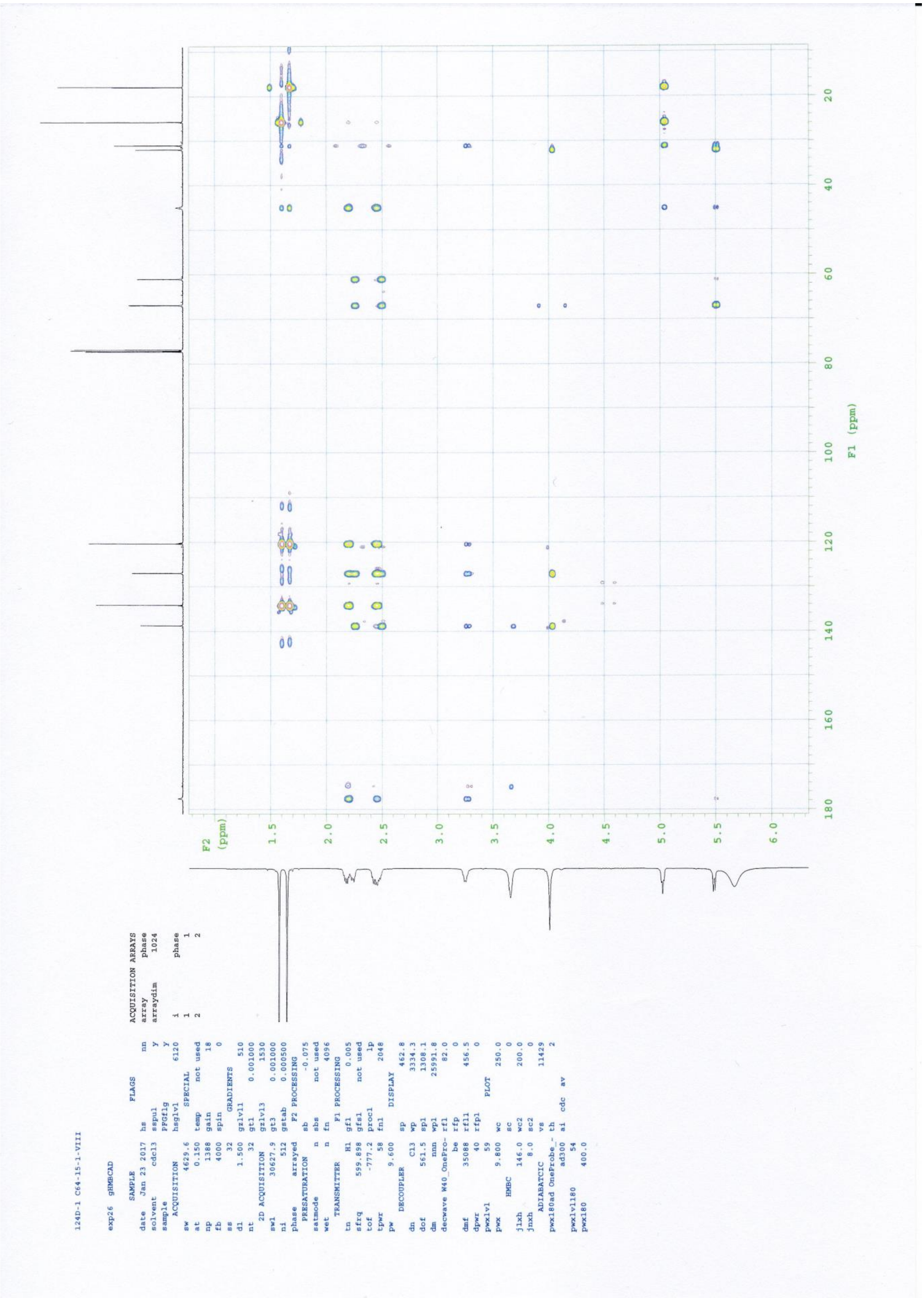

Figure S7 ¹H NMR spectrum of 2 in $CDCl_3$

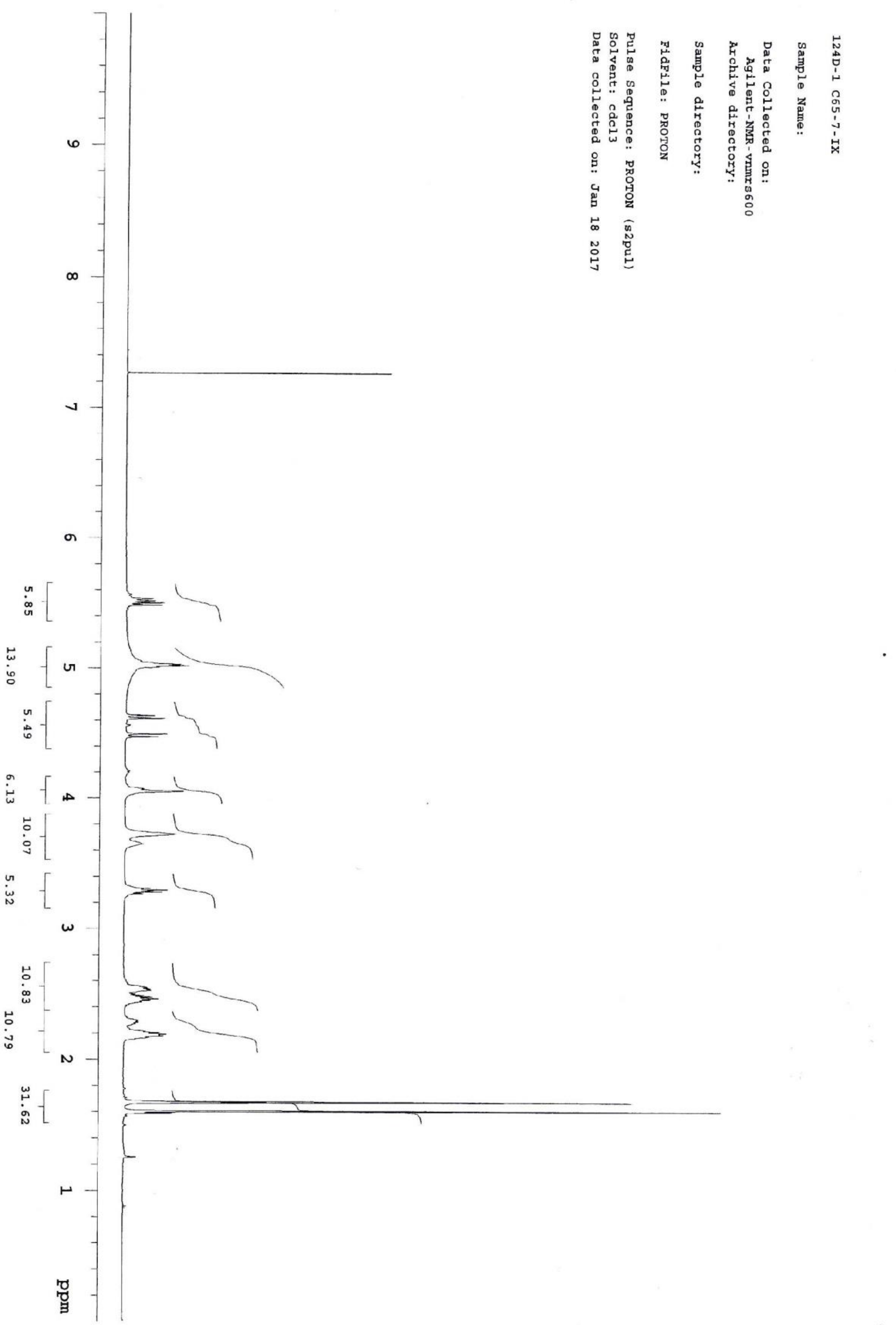

Figure S8 ^{13}C NMR spectrum of **2** in $CDCl_3$

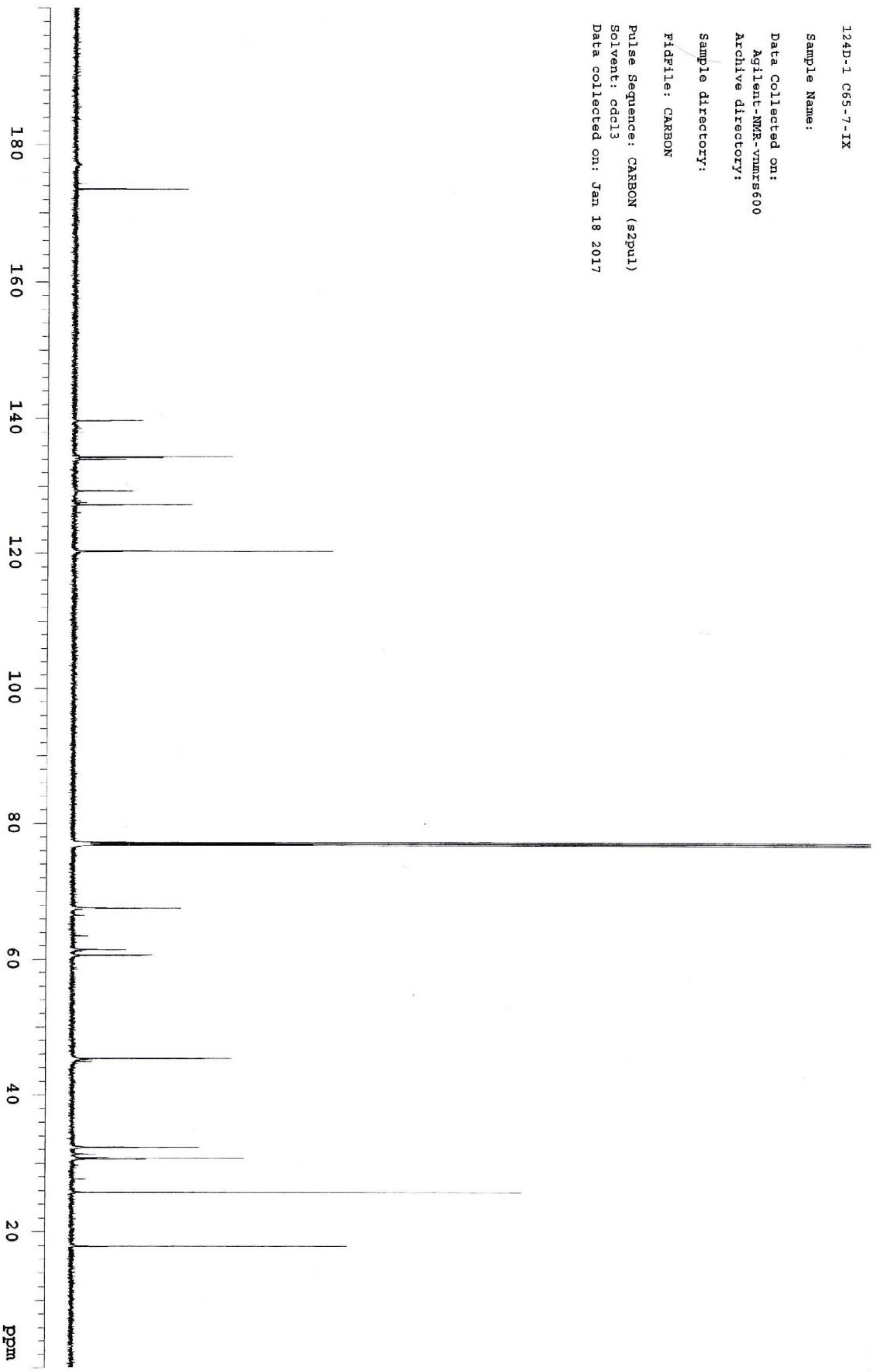

Figure S9 ^{1}H-^{1}H COSY of 2

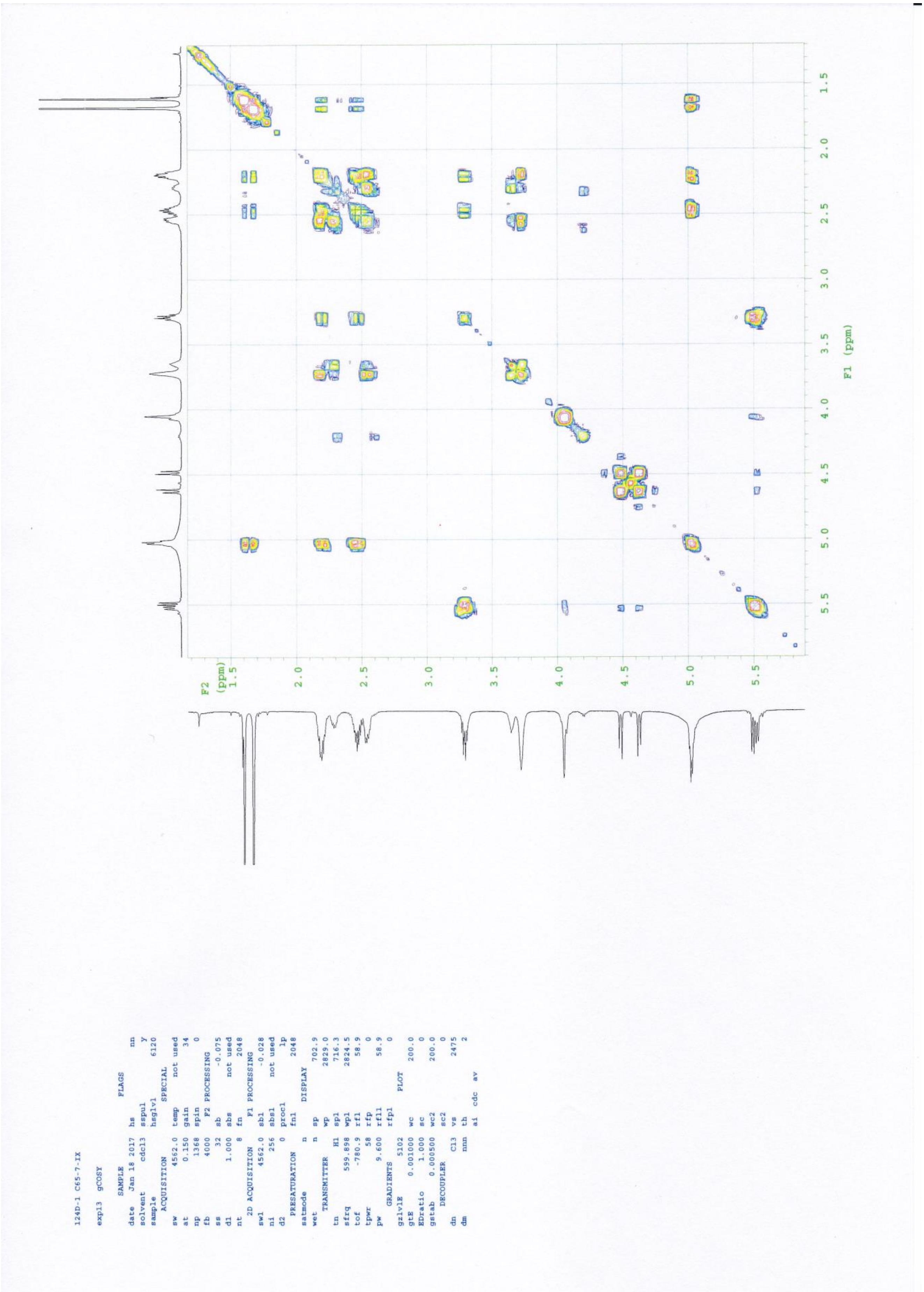

Figure S10 NOESY of 2

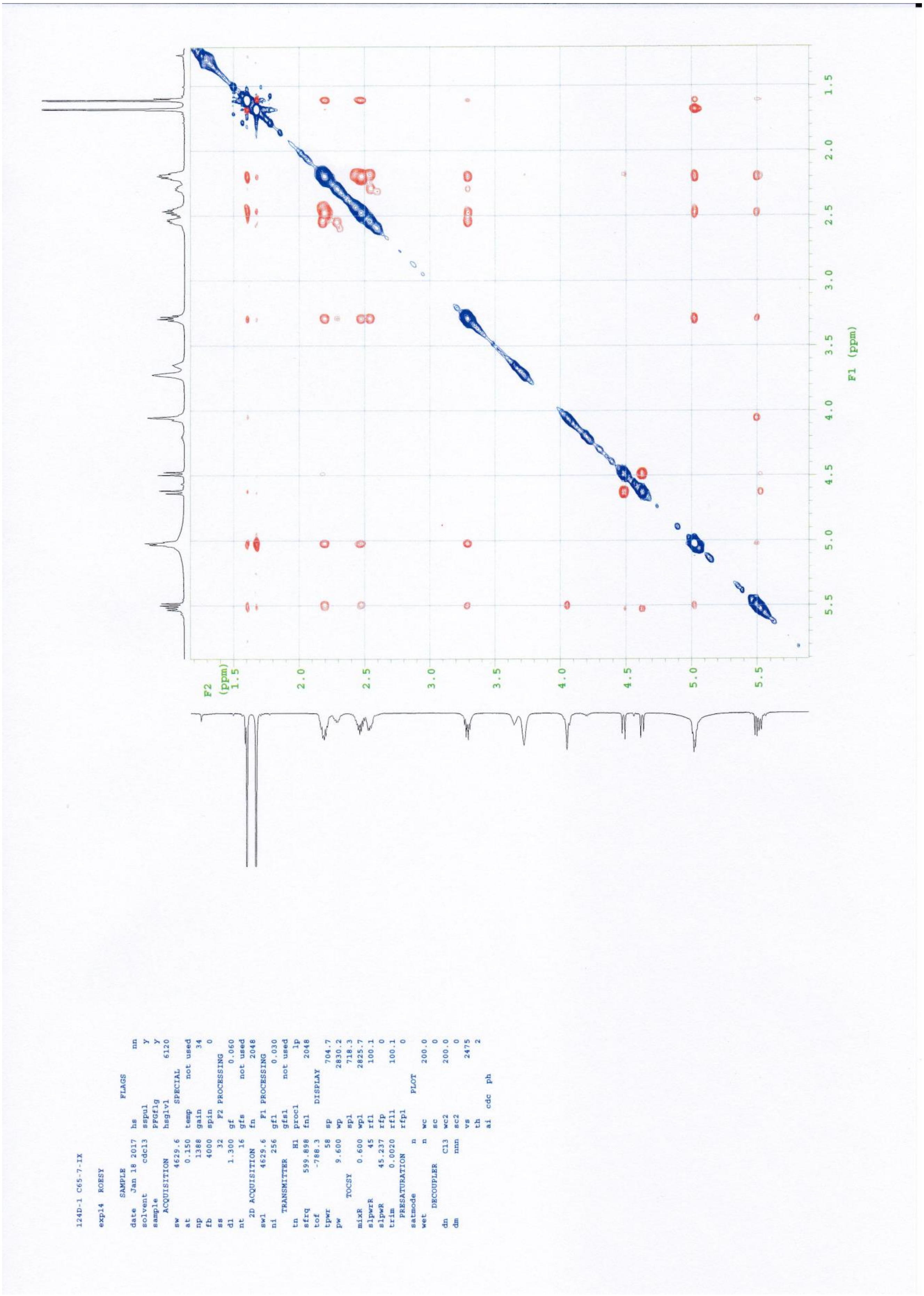

Figure S11 HMQC of 2

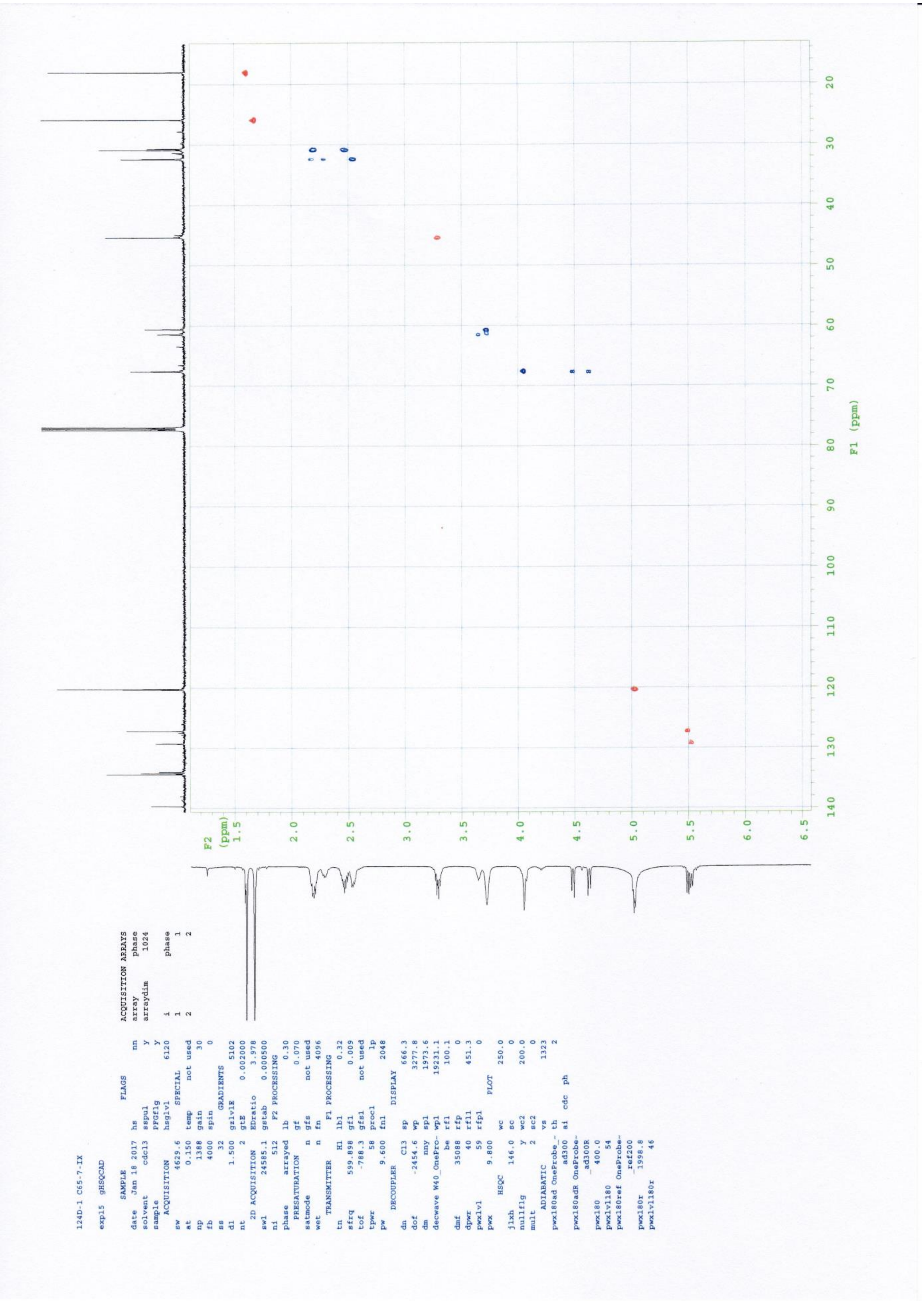

Figure S12 HMBC of 2

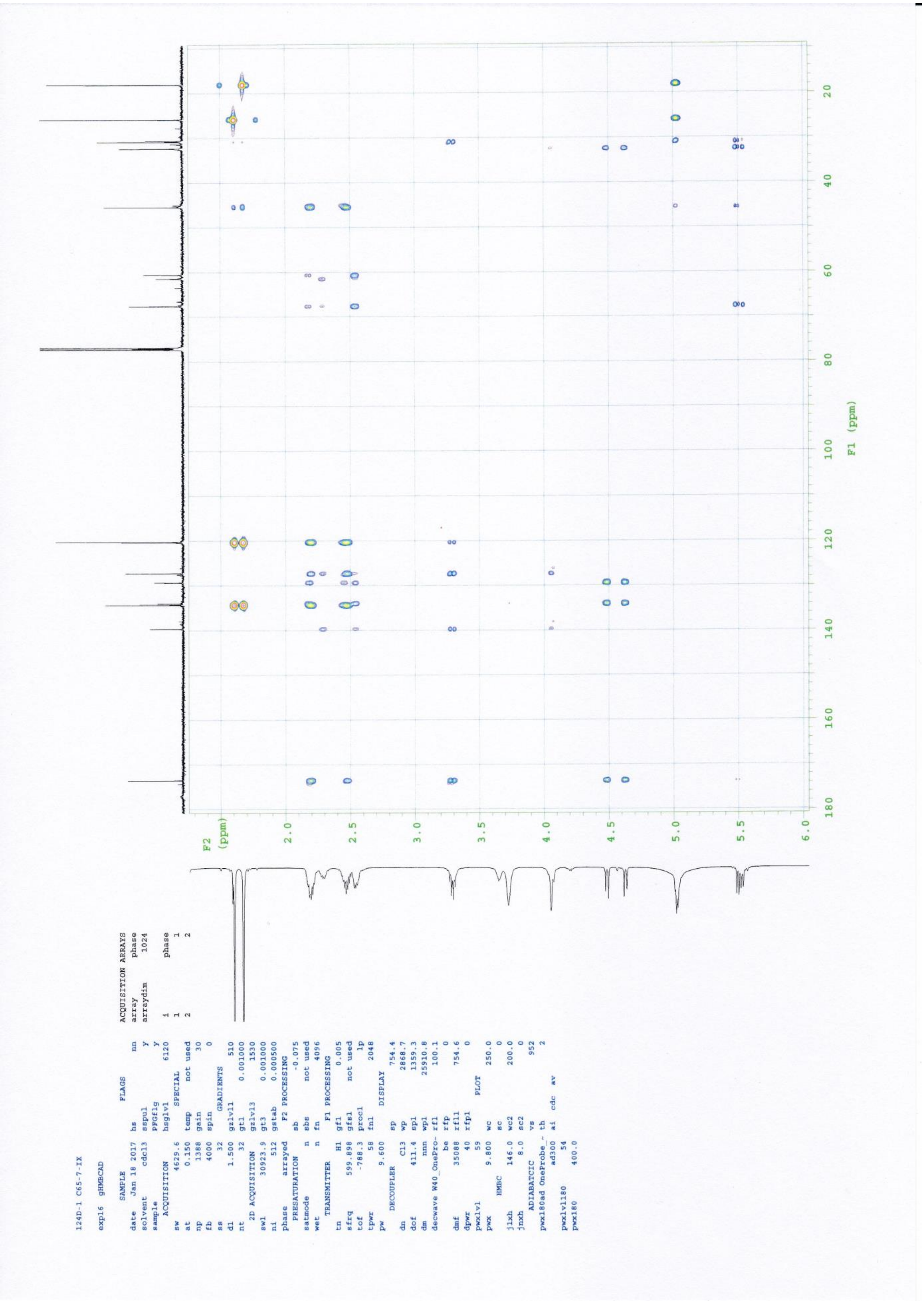

Figure S13 ^{1}H NMR spectrum of 3 in $CDCl_3$

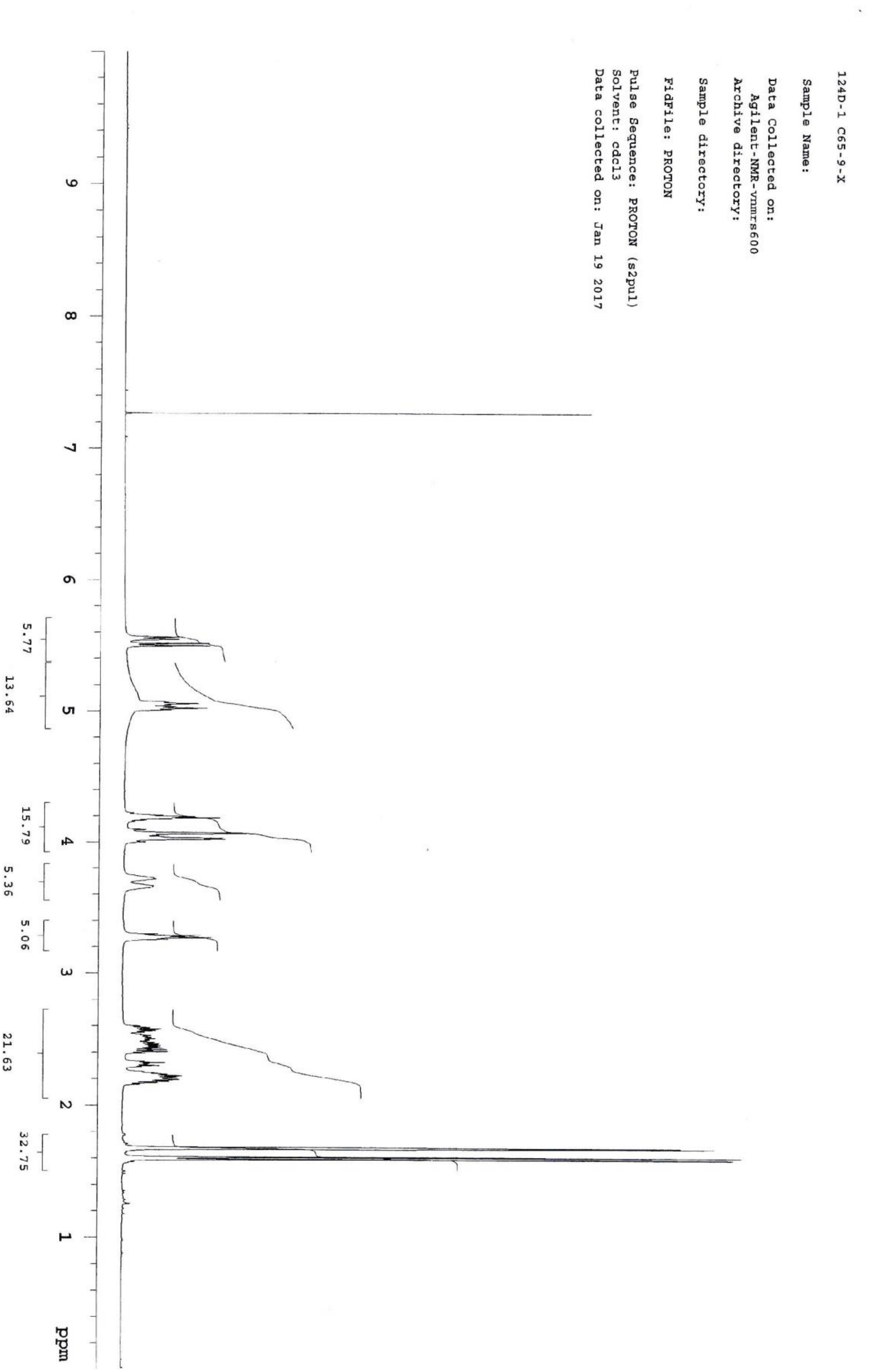

Figure S14 ^{13}C NMR spectrum of 3 in CDCl$_3$

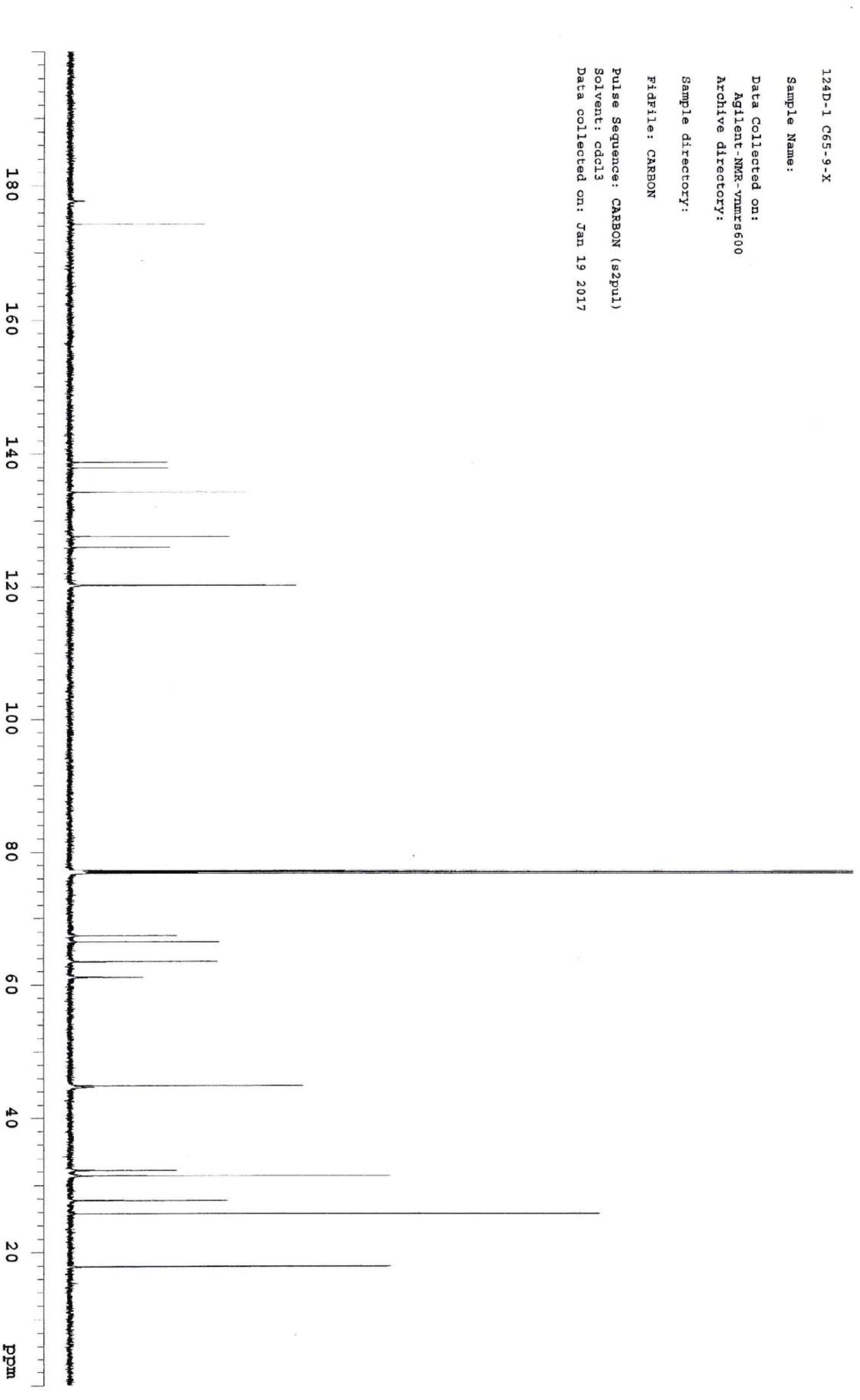

.

Figure S15 ^{1}H-^{1}H COSY of 3

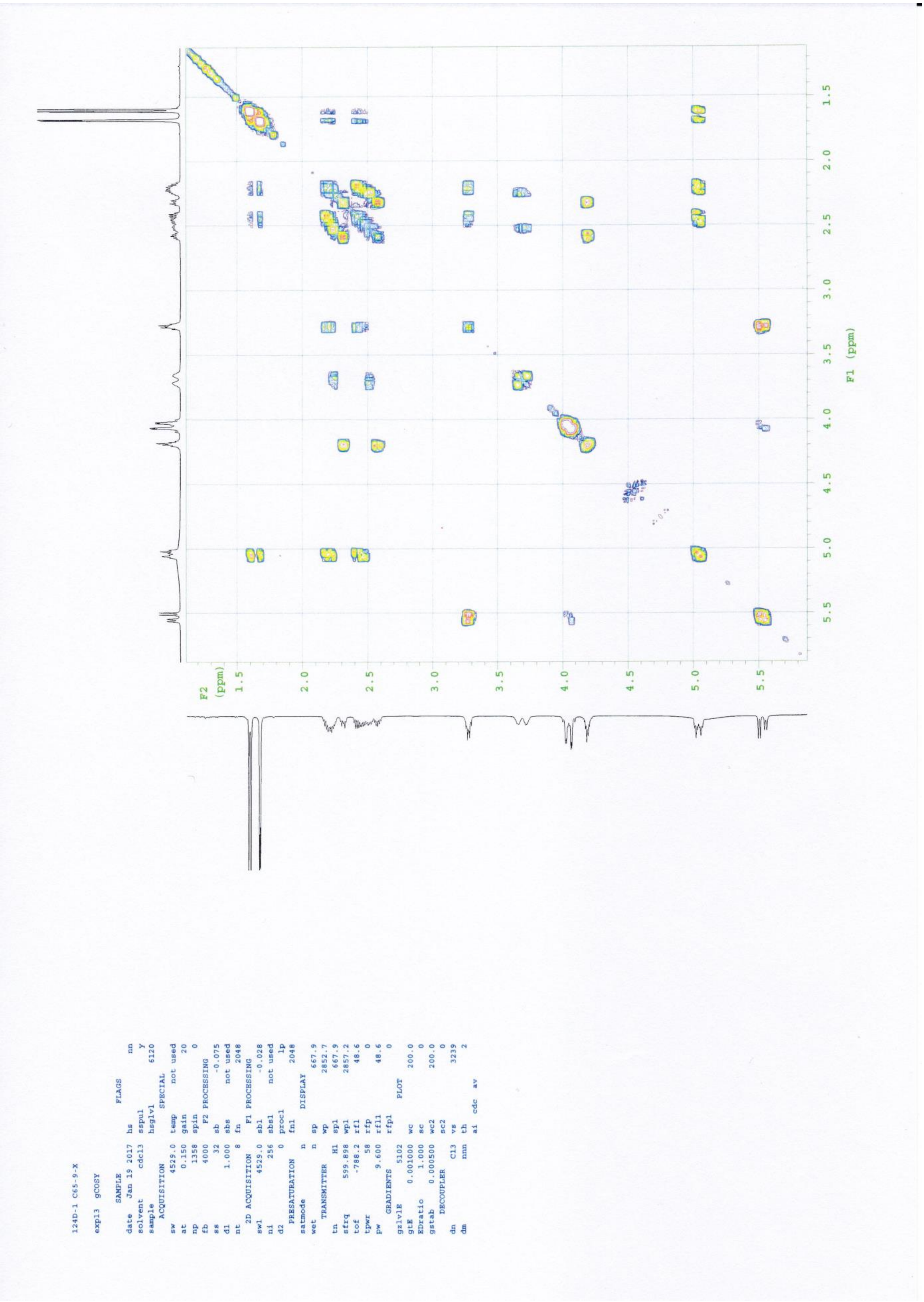

Figure S16 NOESY of 3

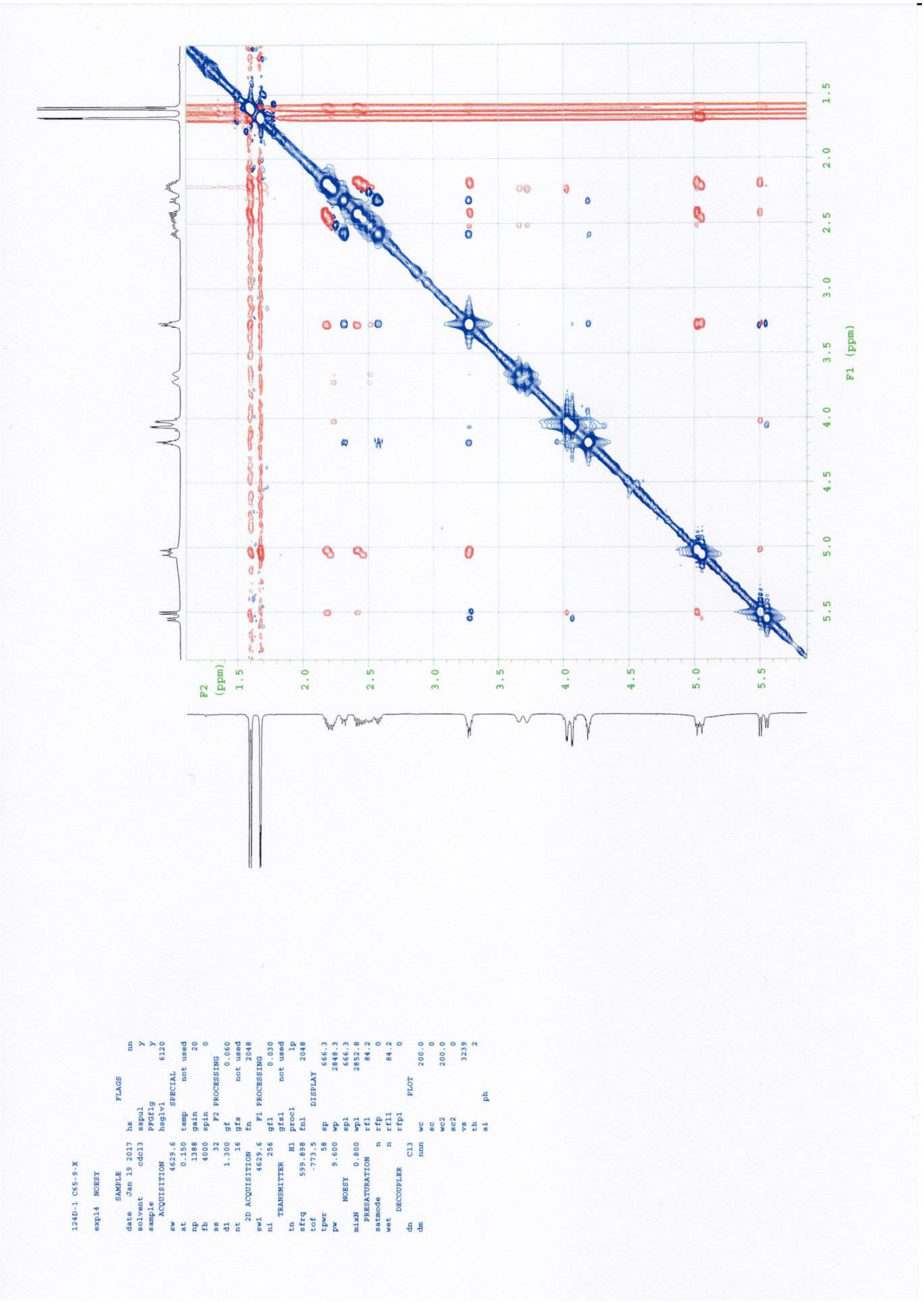

Figure S17 HMQC of 3

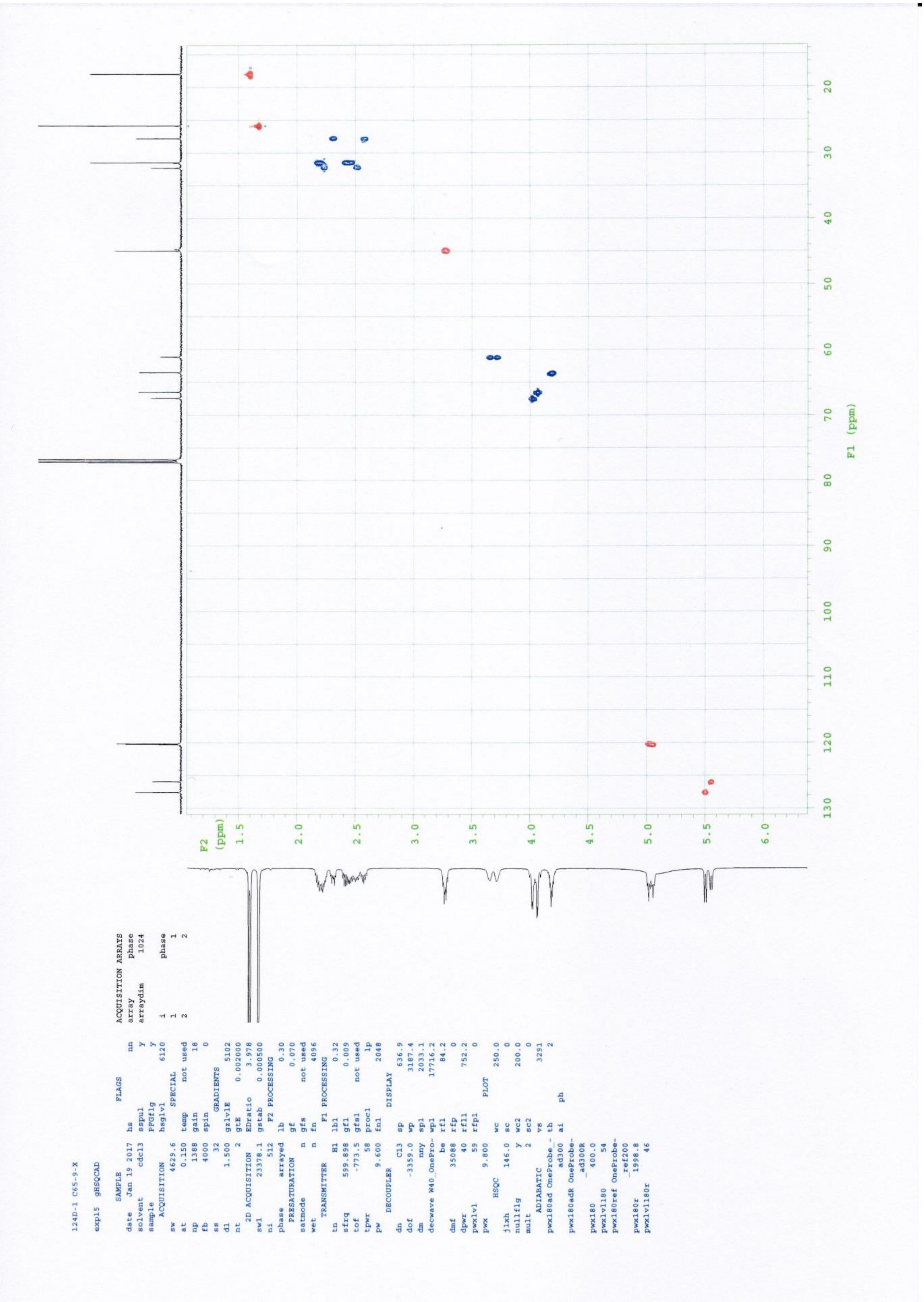

Figure S18 HMBC of 3

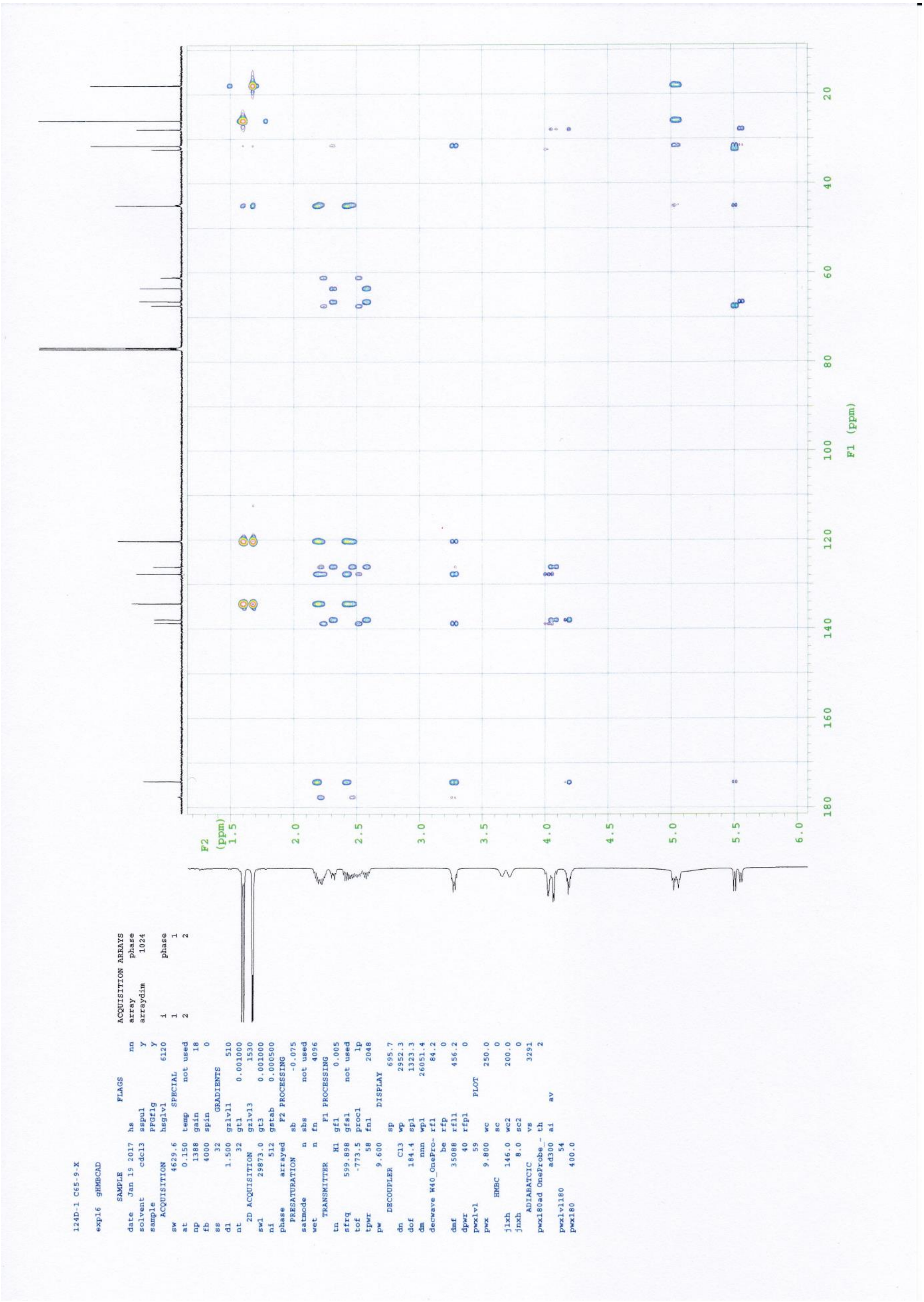

Figure S19 ^{1}H NMR spectra of 4 in $CDCl_3$

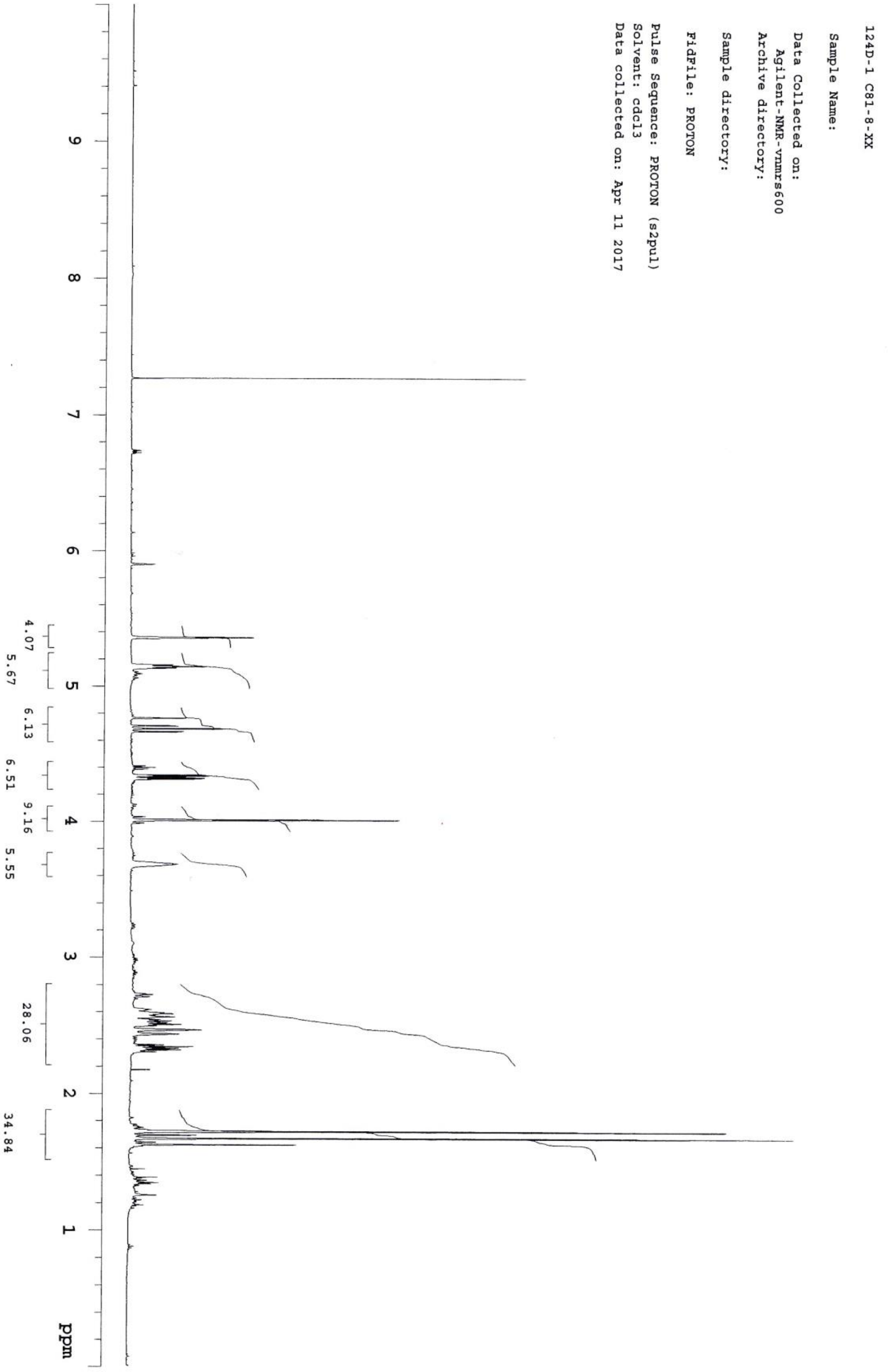

Figure S20 ^{13}C NMR spectra of 4 in $CDCl_3$

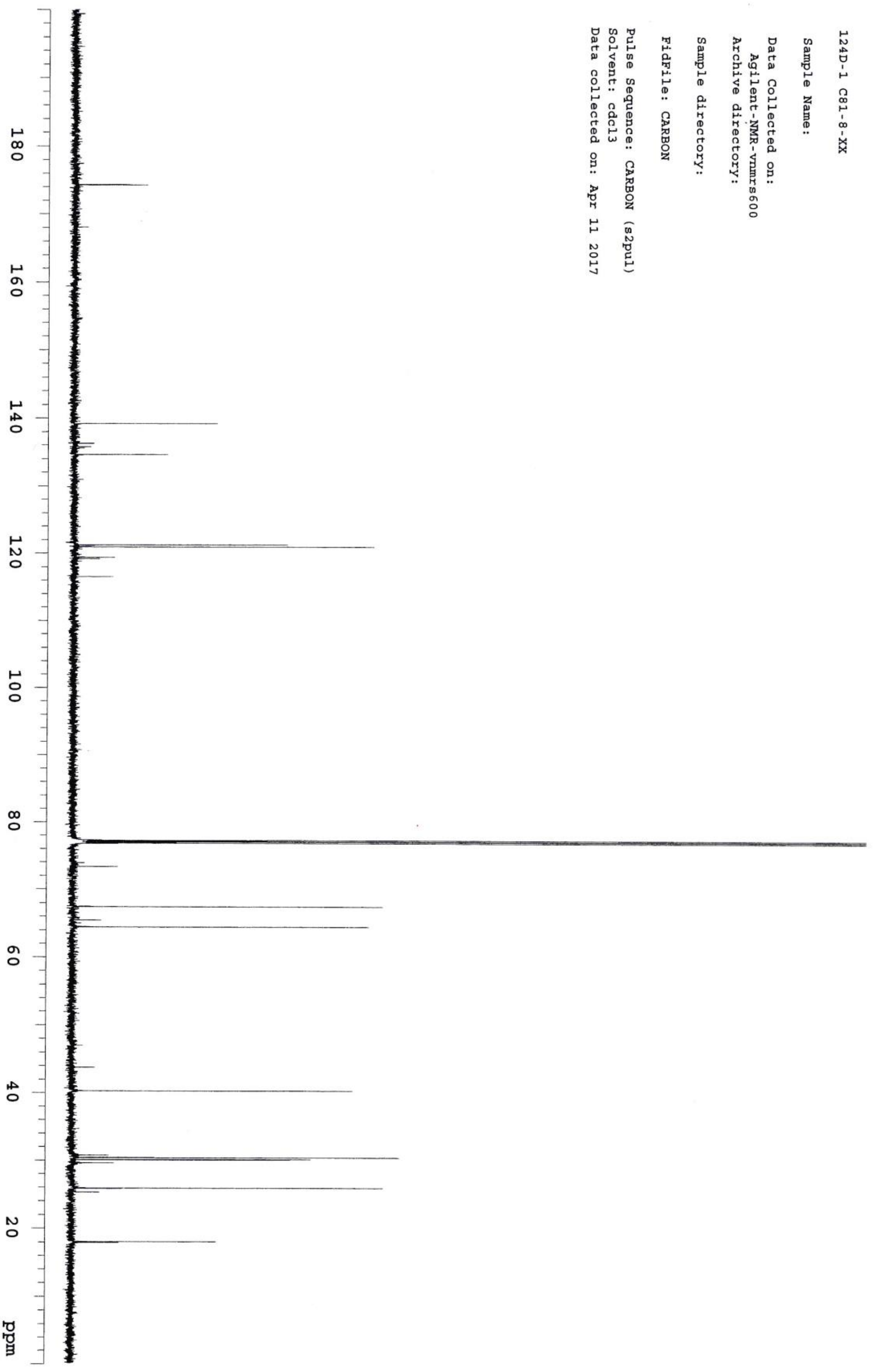

Figure S21 ^{1}H-^{1}H COSY of 4

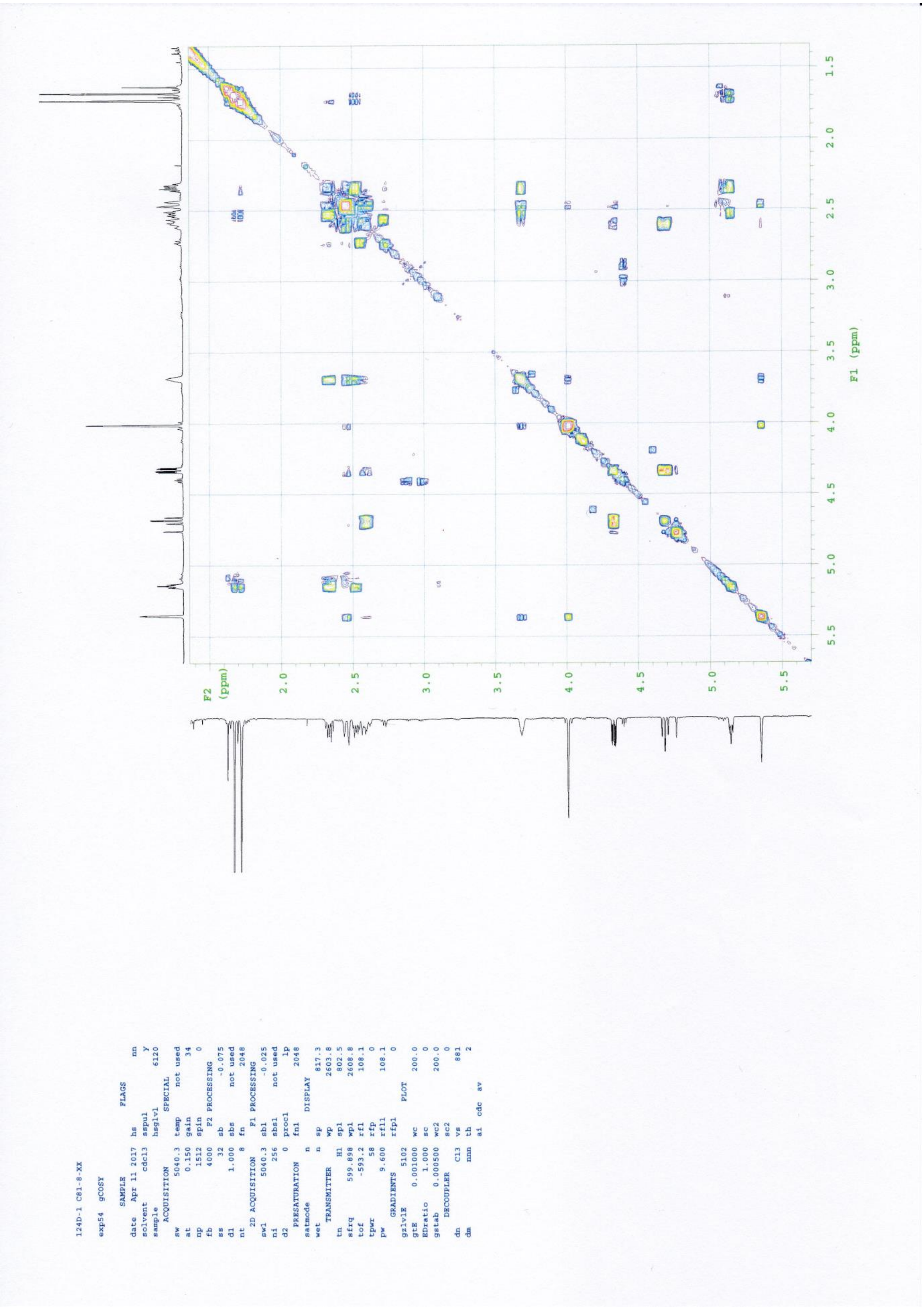

Figure S22 NOESY of 4^+

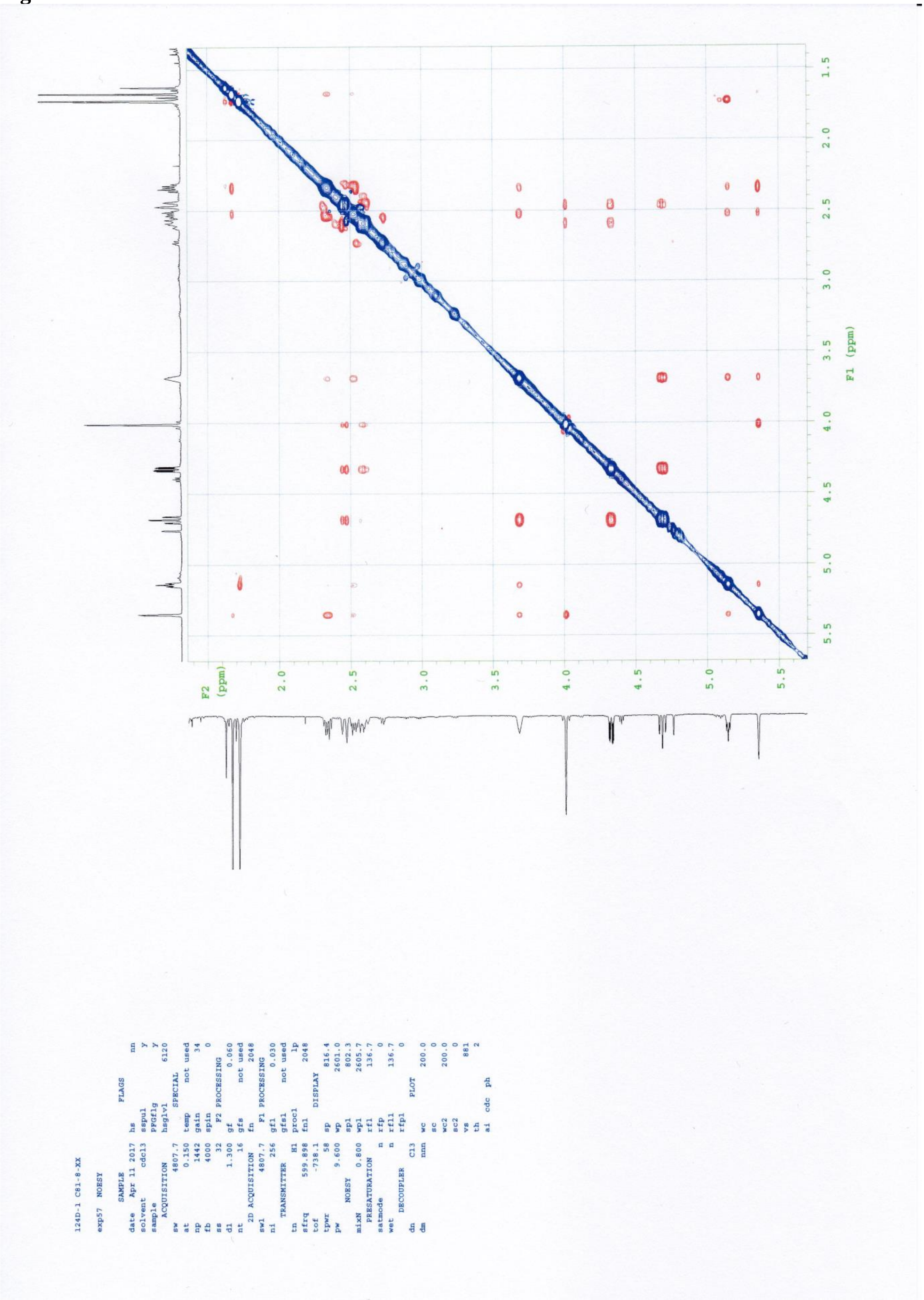

Figure S23 HMQC of 4

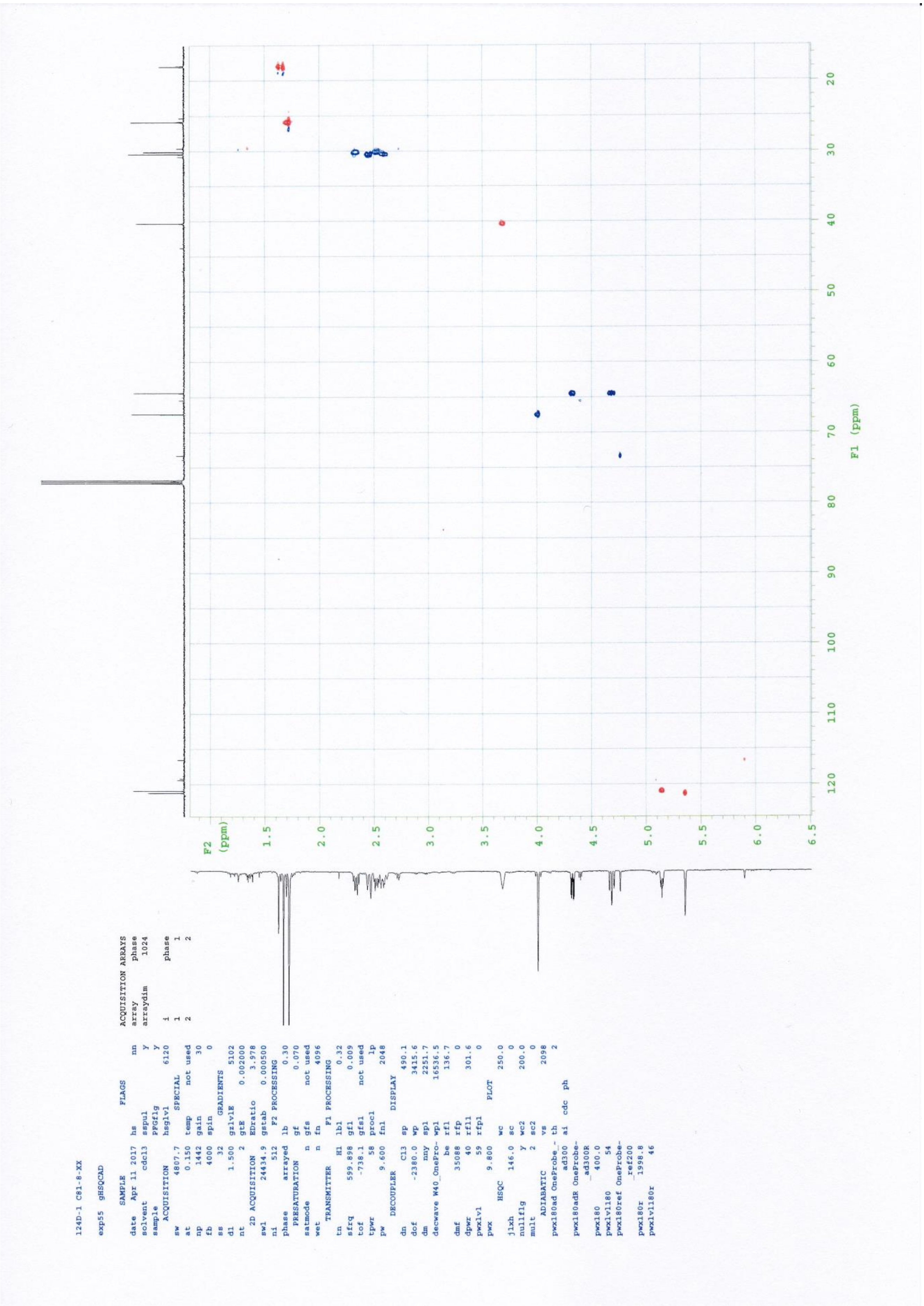

Figure S24 HMBC of 4

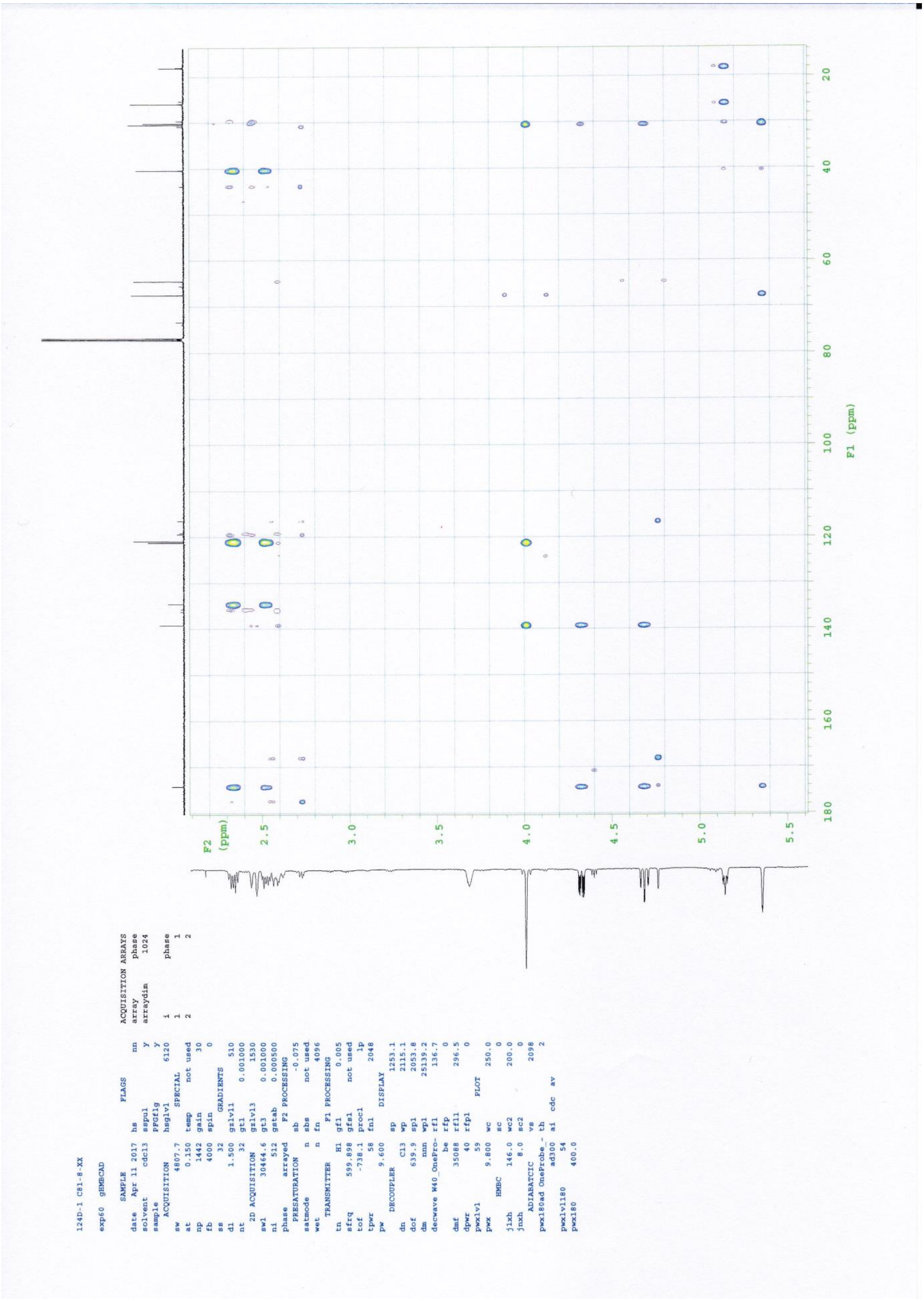

Figure S25 ¹H NMR spectra of 1a in $CDCl_3$

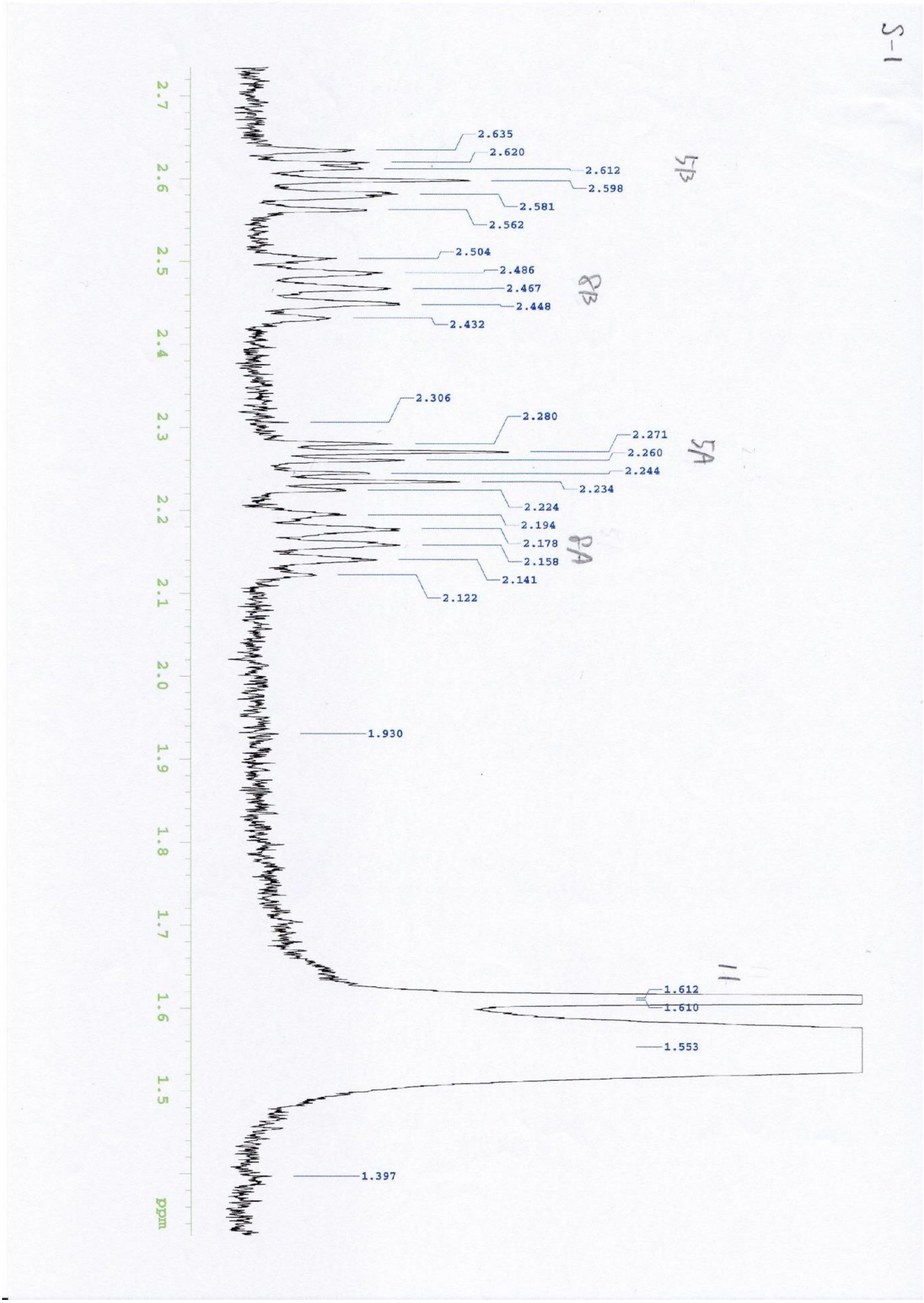

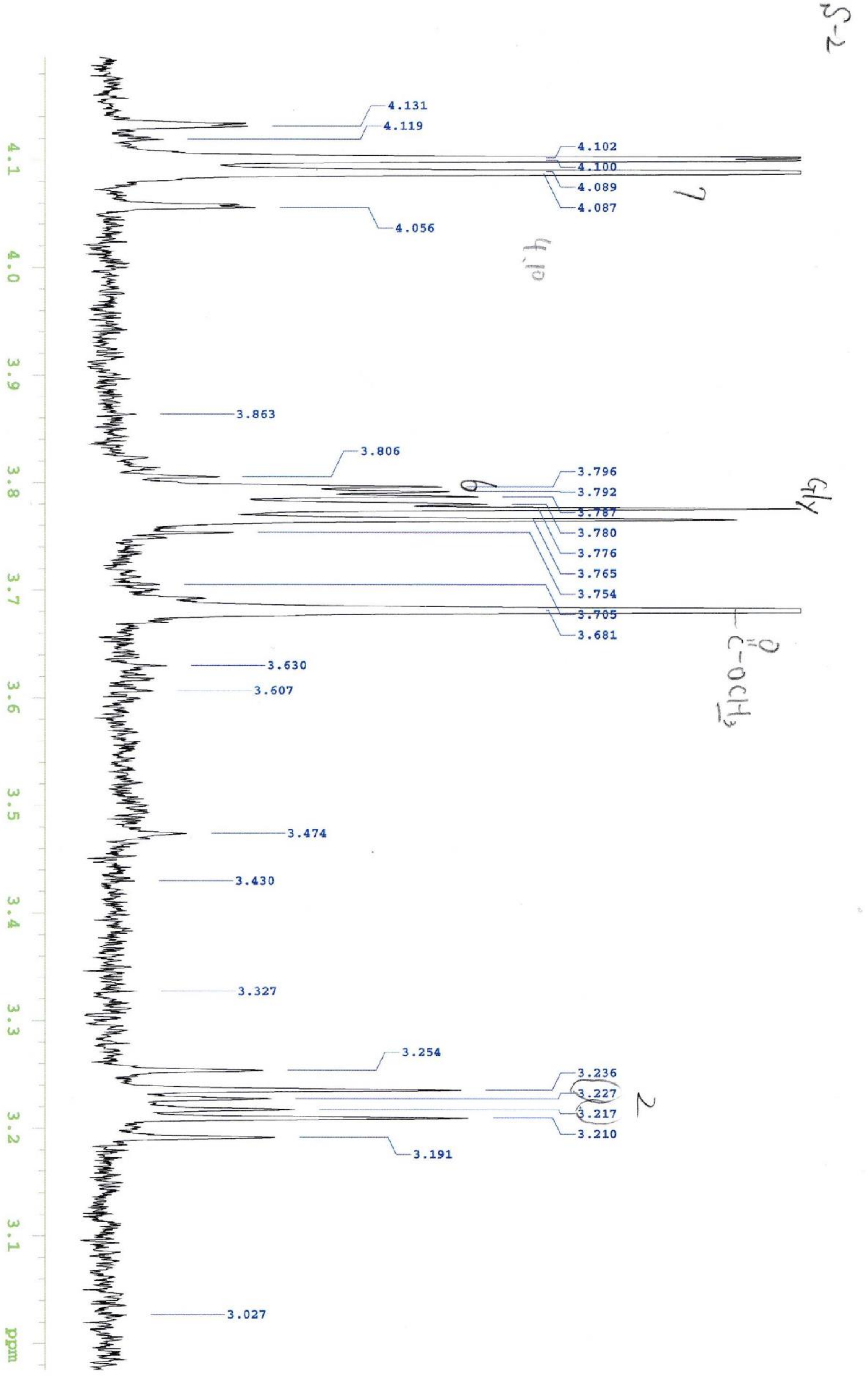
4.131
4.119
4.102
4.100
4.089
4.087
4.056
3.863
3.806
3.796
3.792
3.787
3.780
3.776
3.765
3.754
3.705
3.681
3.630
3.607
3.474
3.430
3.327
3.254
3.236
3.227
3.217
3.210
3.191
3.027
4.1
4.0
3.9
3.8
3.7
3.6
3.5
3.4
3.3
3.2
3.1
ppm
Gly

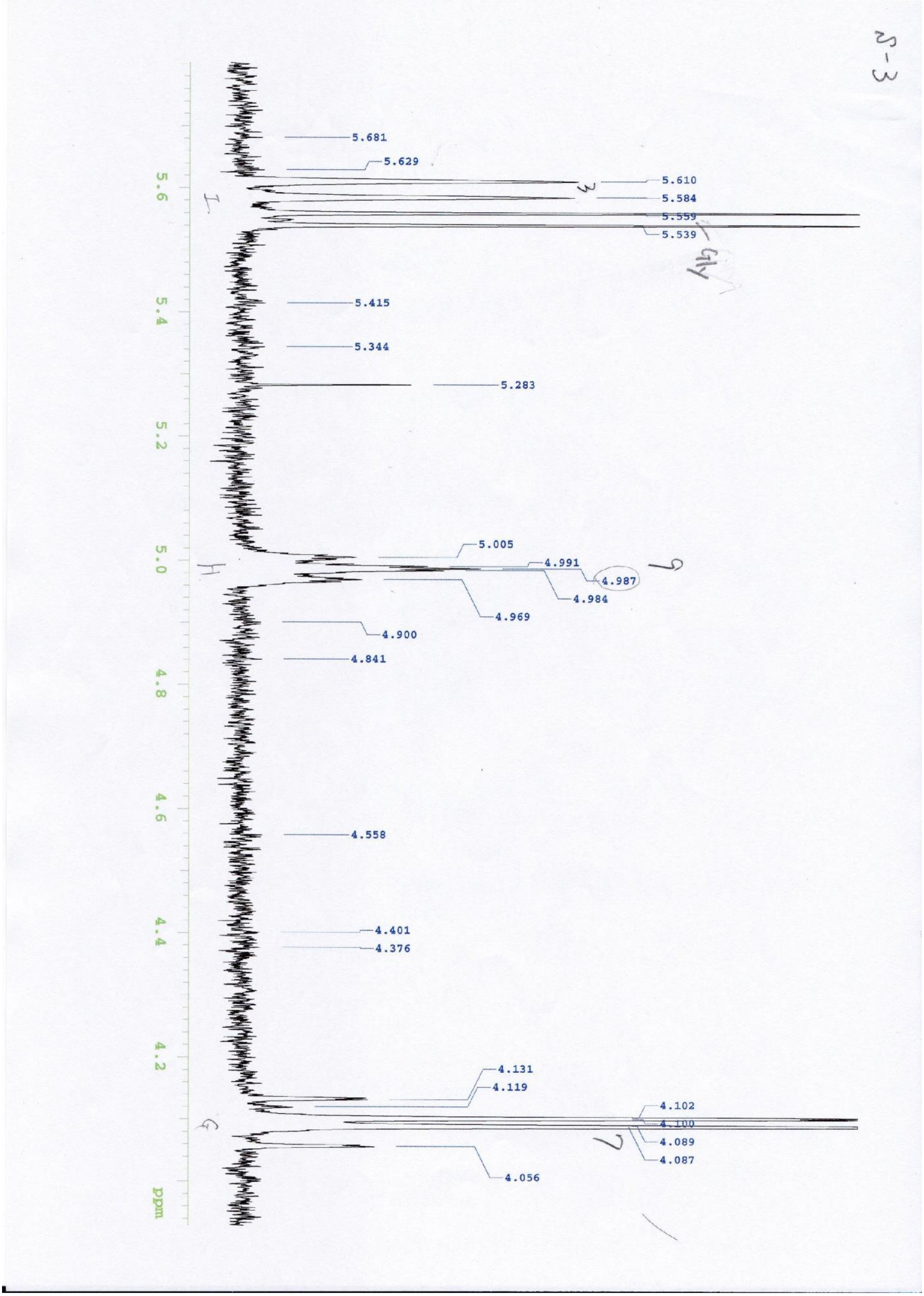
5.681
5.629
5.610
5.584
5.559
5.539
5.415
5.344
5.283
5.005
4.991
4.987
4.984
4.969
4.900
4.841
4.558
4.401
4.376
4.131
4.119
4.102
4.100
4.089
4.087
4.056
5.6
5.4
5.2
5.0
4.8
4.6
4.4
4.2
ppm

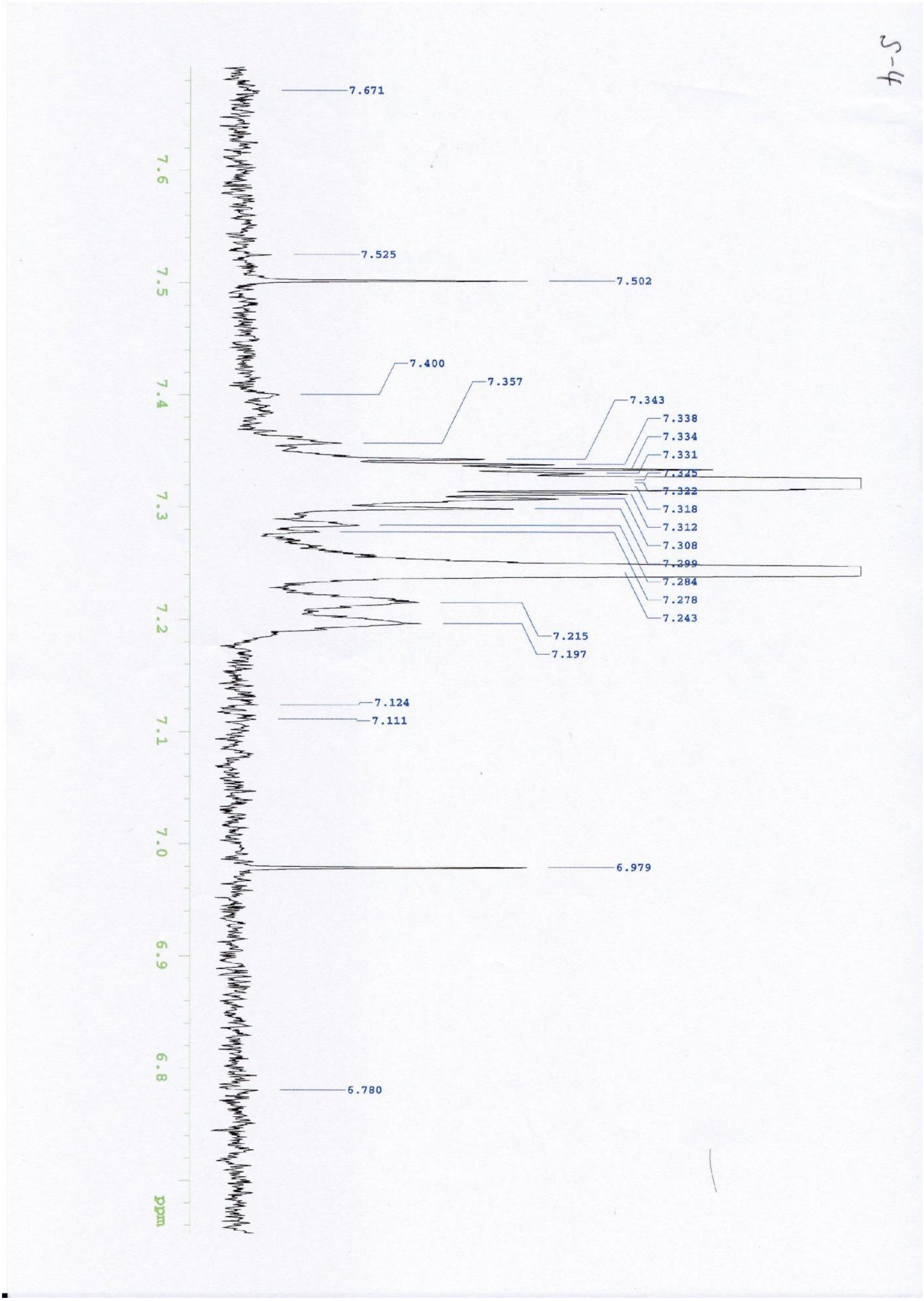
S-4
7.671
7.525
7.502
7.400
7.357
7.343
7.338
7.334
7.331
7.325
7.322
7.318
7.312
7.308
7.299
7.284
7.278
7.243
7.215
7.197
7.124
7.111
6.979
6.780
7.6
7.5
7.4
7.3
7.2
7.1
7.0
6.9
6.8
ppm

Figure S26 ^{1}**H NMR spectra of 2a in** $CDCl_{3+}$

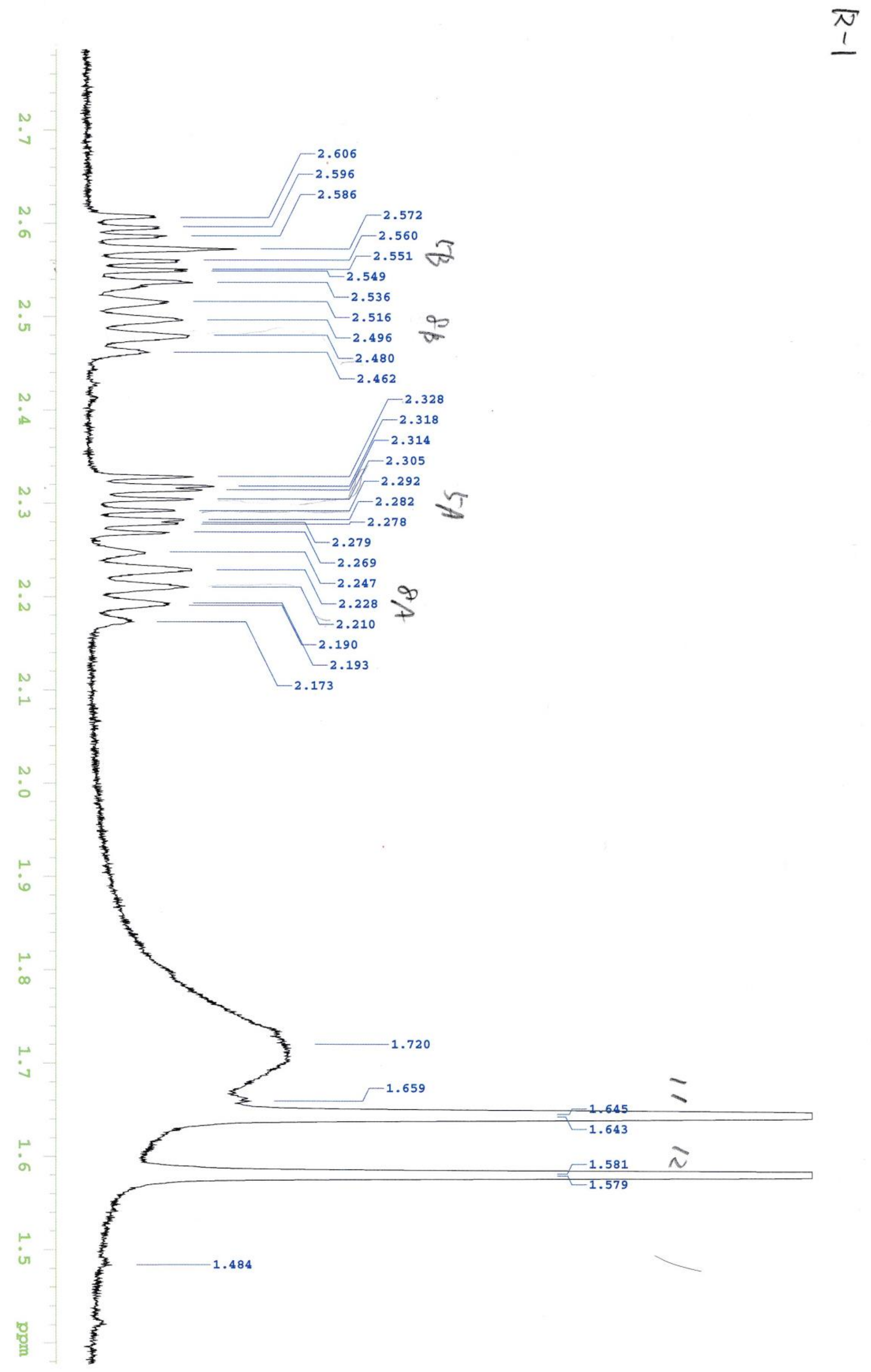

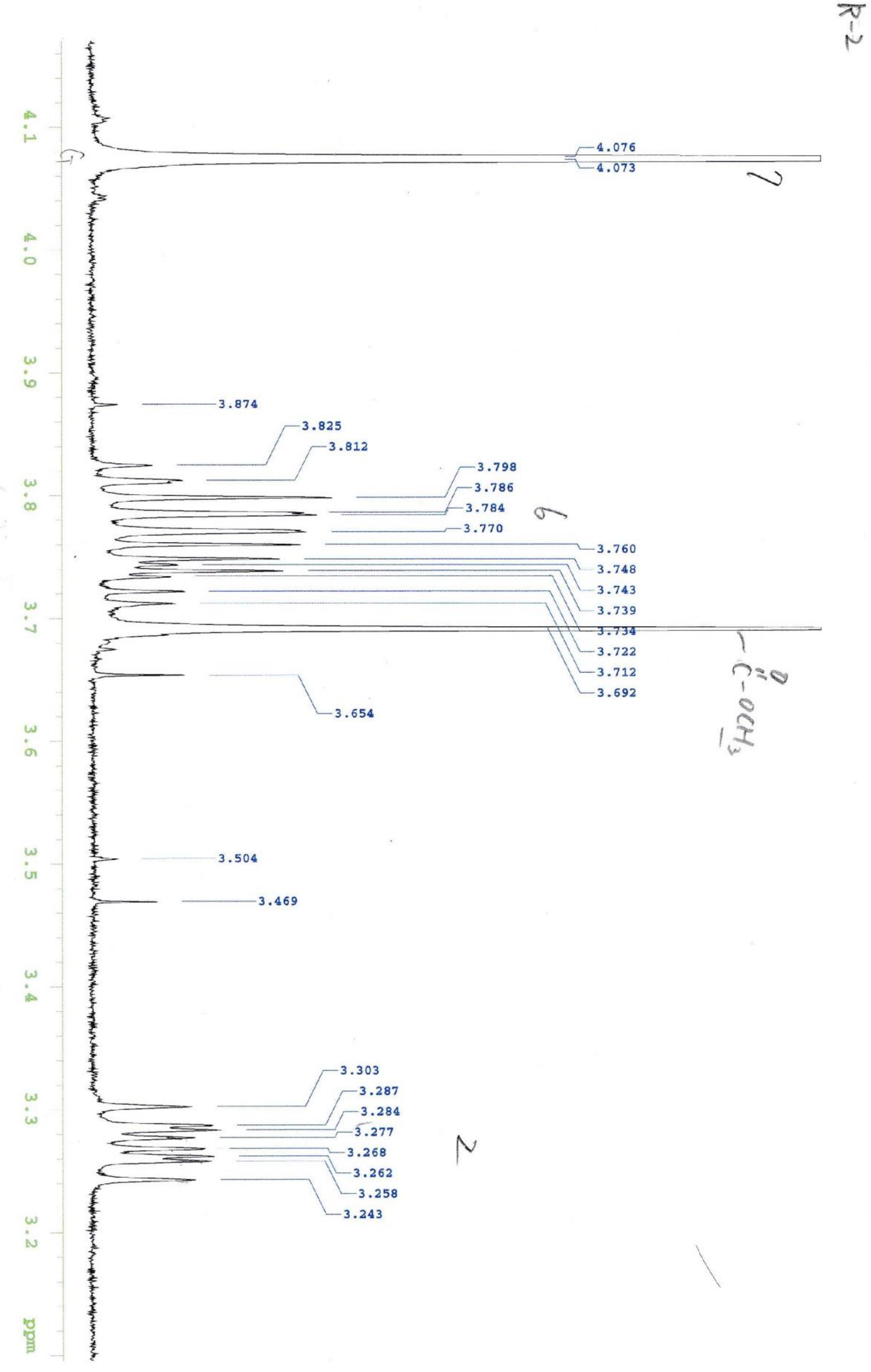
R-2
4.076
4.073
3.874
3.825
3.812
3.798
3.786
3.784
3.770
3.760
3.748
3.743
3.739
3.734
3.722
3.712
3.692
3.654
3.504
3.469
3.303
3.287
3.284
3.277
3.268
3.262
3.258
3.243
4.1
4.0
3.9
3.8
3.7
3.6
3.5
3.4
3.3
3.2
ppm

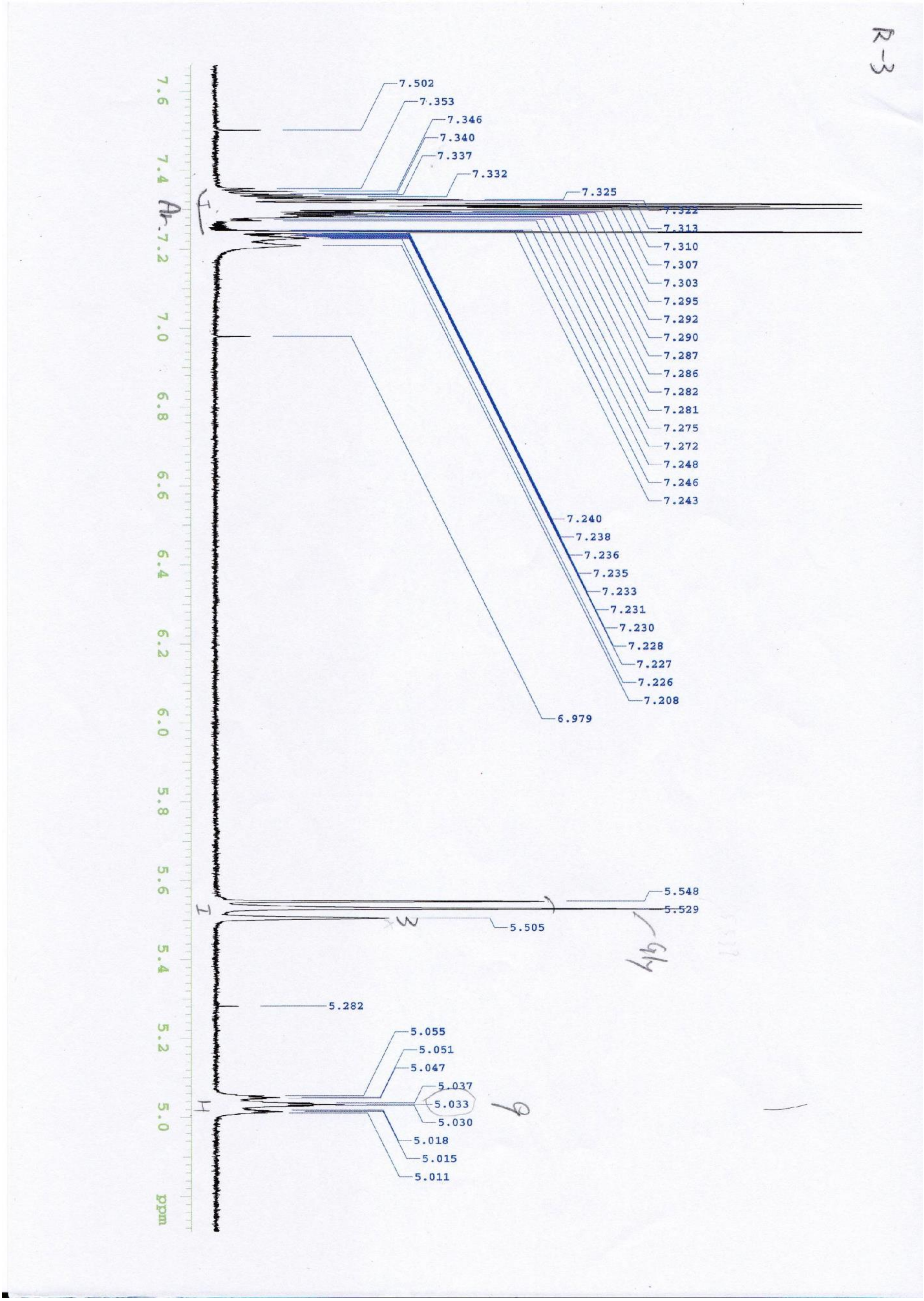
R-3
7.502
7.353
7.346
7.340
7.337
7.332
7.325
7.322
7.313
7.310
7.307
7.303
7.295
7.292
7.290
7.287
7.286
7.282
7.281
7.275
7.272
7.248
7.246
7.243
7.240
7.238
7.236
7.235
7.233
7.231
7.230
7.228
7.227
7.226
7.208
6.979
5.548
5.529
5.505
5.282
5.055
5.051
5.047
5.037
5.033
5.030
5.018
5.015
5.011
7.6
7.4
7.2
7.0
6.8
6.6
6.4
6.2
6.0
5.8
5.6
5.4
5.2
5.0
ppm

Figure S27 ^{1}H NMR spectra of 2b in $CDCl_3$

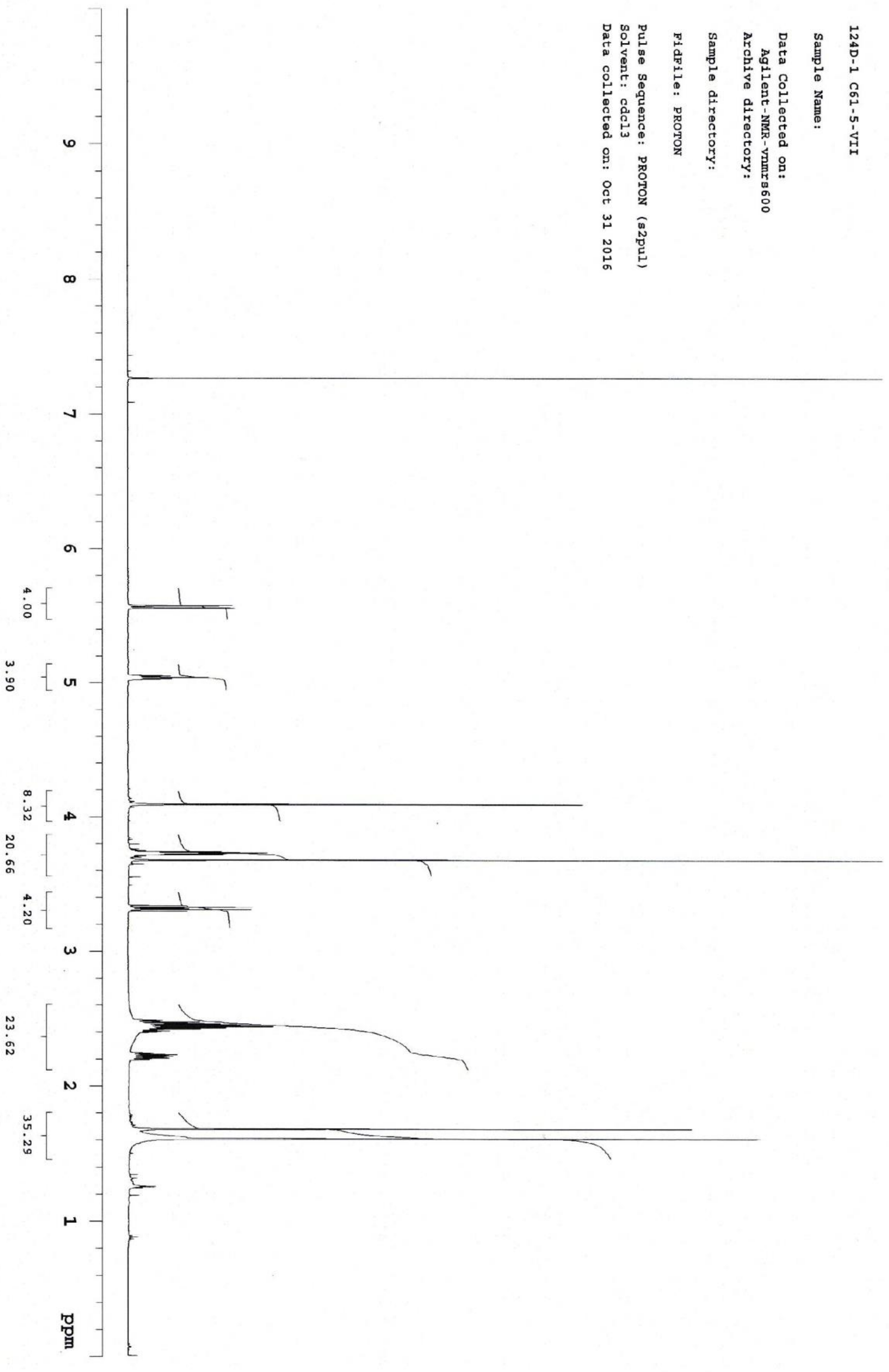

-
